# Regional variations in discrete collagen fibre mechanics within intact intervertebral disc resolved using synchrotron computed tomography and digital volume correlation

**DOI:** 10.1016/j.actbio.2021.10.012

**Published:** 2022-01-15

**Authors:** C.M. Disney, J. Mo, A. Eckersley, A.J. Bodey, J.A. Hoyland, M.J. Sherratt, A.A. Pitsillides, P.D. Lee, B.K. Bay

**Affiliations:** a Mechanical Engineering, University College London, UK; b School of Biological Sciences, The University of Manchester, UK; c Diamond Light Source, Oxfordshire, UK; d NIHR Manchester Biomedical Research Centre, Central Manchester Foundation Trust, Manchester Academic Health Science Centre, Manchester, UK; e Department of Comparative Biomedical Sciences, Skeletal Biology Group, Royal Veterinary College, London, UK; f School of Mechanical, Industrial and Manufacturing Engineering, Oregon State University, OR, USA

**Keywords:** Intervertebral Disc, Synchrotron Tomography, Digital Volume Correlation, Collagen Fibre Organisations

## Abstract

Many soft tissues, such as the intervertebral disc (IVD), have a hierarchical fibrous composite structure which suffers from regional damage. We hypothesise that these tissue regions have distinct, inherent fibre structure and structural response upon loading. Here we used synchrotron computed tomography (sCT) to resolve collagen fibre bundles (∼5μm width) in 3D throughout an intact native rat lumbar IVD under increasing compressive load. Using intact samples meant that tissue boundaries (such as endplate-disc or nucleus-annulus) and residual strain were preserved; this is vital for characterising both the inherent structure and structural changes upon loading in tissue regions functioning in a near-native environment. Nano-scale displacement measurements along >10,000 individual fibres were tracked, and fibre orientation, curvature and strain changes were compared between the posterior-lateral region and the anterior region. These methods can be widely applied to other soft tissues, to identify fibre structures which cause tissue regions to be more susceptible to injury and degeneration. Our results demonstrate for the first time that highly-localised changes in fibre orientation, curvature and strain indicate differences in regional strain transfer and mechanical function (e.g. tissue compliance). This included decreased fibre reorientation at higher loads, specific tissue morphology which reduced capacity for flexibility and high strain at the disc-endplate boundary.

**Statement of significance:**

The analyses presented here are applicable to many collagenous soft tissues which suffer from regional damage. We aimed to investigate regional intervertebral disc (IVD) structural and functional differences by characterising collagen fibre architecture and linking specific fibre- and tissue-level deformation behaviours. Synchrotron CT provided the first demonstration of tracking discrete fibres in 3D within an intact IVD. Detailed analysis of regions was performed using over 200k points, spaced every 8 μm along 10k individual fibres. Such comprehensive structural characterisation is significant in informing future computational models. Morphological indicators of tissue compliance (change in fibre curvature and orientation) and fibre strain measurements revealed localised and regional differences in tissue behaviour.

## Graphical abstract

**Figure:**
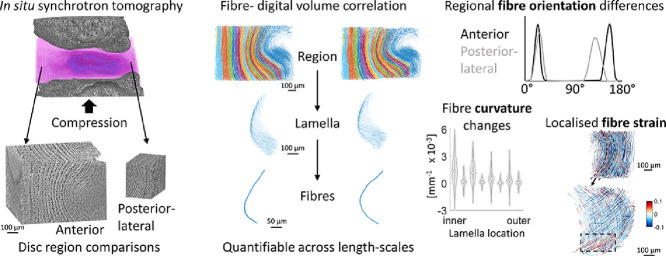

## 1 Introduction

Many soft tissues contain a hierarchical fibrous composite microstructure that is vital for their functional competence. The non-linear and anisotropic mechanical properties of these composites allow flexibility as fibres reorganise at low loads, and high stiffness as fibres are strained at high loads. Such properties are highly reliant on inherent and load-induced tissue fibre organisation. Thus, collagen fibres in tendon are aligned and prestressed to support rapid uptake of tensile loads [1]; whereas alternating layers of angulated fibres engender a controlled compliance ahead of peak load and pressurisation in arteries and intervertebral discs [2,3]. These fibrous tissues, however, are known to exhibit profound regional susceptibility to failure; with patellar tendinopathy mostly localised to the proximal and posterior portion [4], aneurysms targeted to the abdominal section of the aorta [5] and degeneration in the intervertebral disc - the focus herein – concentrated to its posterior-lateral region [6,7]. Despite this wealth of clinical evidence, it remains unknown whether there is a regional distinction in collagen fibre architecture and whether there is region specific fibre- and tissue-level deformation behaviours when subjected to mechanical load.

**Fig. 1:**
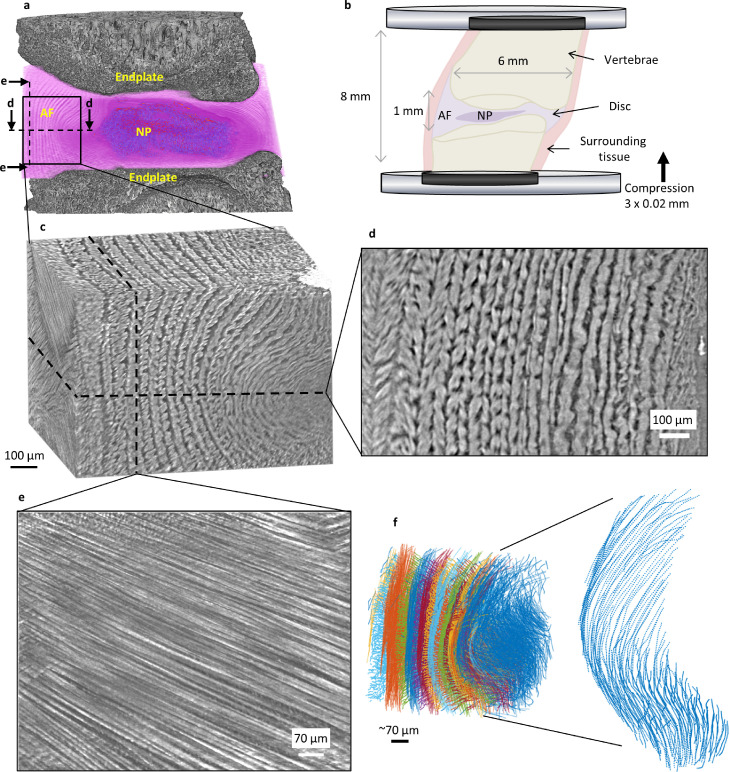
**Collagen bundle architecture of a whole intervertebral disc under compression resolved using synchrotron tomography.** (**a)** Rendered tomogram of spine segment clipped through the sagittal plane. (**b)***In situ* imaging setup. (**c)** Anterior region of interest rendering. (**d)** Transverse slice across anterior volume showing lamellae across the full thickness of the AF are resolved. (**e)** Slice across anterior volume showing collagen fibre bundles in a single lamella. (**f)** >10k individual collagen fibre bundles traced across the anterior volume. Fibres are defined by closely spaced points (8 µm) and labelled by lamella.

Intervertebral disc (IVD) degeneration is the leading cause of low back pain [8] and a significant socio-economic healthcare burden [9]. The IVD has a central gel-like nucleus pulposus (NP) surrounded by annulus fibrosus (AF; Fig. 1a) which attaches to the vertebral endplates. The AF has a hierarchical lamellar structure consisting of layered dense bundles of aligned type I collagen, with fibre orientation which alternates in adjacent lamellae [2]. The unloaded IVD has a residual strain which is considered vital for normal mechanical function and for protection against peak stresses under high loads [10,11]. As the disc is loaded, NP pressure increases and is contained by the AF. AF collagen fibres are recruited in response to the increasing tensile hoop stresses by re-orientating toward the transverse plane [12].

AF fibre orientation, residual strain and tissue mechanical properties are known to vary across different IVD regions [11,13,14]. Crucially, clinical findings indicate higher incidence of AF damage/herniation in the posterior regions [6,7], associated with disrupted collagen fibre organisation and loss of residual strain [10] and yet the regional architectural differences in fibre orientation, curvature and organisation into lamellae, remain somewhat elusive. Moreover, the impact of these architectural differences on regional mechanical behaviours, including the essential transfer of load-induced deformation across hierarchical-scales (from change in AF tissue microstructure to change in fibre morphology and strain) are ill-defined.

We hypothesise that tissue regions have distinct, inherent structure and structural response upon loading. Exposing these structures requires interrogation of tissue architecture at fibre-level (micrometre) resolution in 3D within intact, entire IVD under increasing load, that will additionally allow regional comparisons. Whilst strain measurements have been made on dissected IVD segments they are typically limited to only a restricted number of fibres on the sample surface [12,15,16]. Dissecting samples for these studies also involves detaching endplates, cutting through AF and removing the swelling pressure from the NP, which means that residual strain and complex boundary conditions important for normal function are no longer preserved [10,17,18]. This emphasises the significance of characterising IVD fibre architecture and deformation in intact samples, ideally assessing many fibres at high spatial resolution. Although there have been recent advances in ultrahigh field MRI imaging, achieving 100 µm resolution, these studies are yet to resolve entire disc microstructure at fibre resolution which is required for complete regional comparisons [19,20].

Here, we have resolved 3D fibre architecture across the full AF width of an intact rat IVD under varying levels of compression using high-resolution phase-contrast x-ray synchrotron computed tomography (PC-sCT). X-ray micro-tomography is commonly used to resolve microstructure in intact tissue samples [21], [22], [23], [24], but soft tissues generally have poor tomographic imaging characteristics due to low x-ray absorption contrast and the potential for deformation during imaging. We have exploited previous findings which have shown that in-line PC-sCT can resolve AF fibre architecture in native, unfixed, unstained IVD [25]. This approach, also makes it possible to track micro-scale tissue displacements by digital volume correlation (DVC) and therefore enables the regional mapping of strain patterns in the AF of intact IVD segments under compression [26]. However, mapping or continuum measurement of displacements and strain does not provide information on changes in fibre architecture or fibre strain upon loading.

The improved resolution of whole IVD in this study has allowed for high precision tracking of over ten thousand individual collagen fibres per region across the full AF width to specific lamellae using DVC. This enabled highly localised measurement of fibre-level orientation, curvature and strain to be related to tissue function and allowed powerful comparisons across length scales in IVD regions. This revealed notable regional differences in the inherent fibre architecture in the IVD, which influenced strain transfer through the AF, suggesting divergent tissue function between compliance and load transfer. Thus, compliant fibre architecture, demonstrated by changes in tissue morphology and fibre reorientation, showed shifting balance with load transfer behaviour in IVD regions vulnerable to failure.

## 2 Materials and methods

### 2.1 Tissue and materials

Male 8-week-old Sprague Dawley rats (University of Manchester Biological Services Facility) were sacrificed by carbon dioxide inhalation following the University's Animal Research Policy and the UK Animal (Scientific Procedures) Act 1986. Spines were dissected *en bloc* and snap frozen in liquid nitrogen. Samples were stored at -80°C and thawed at room temperature before use. Angled cuts were made through L4/L3 and L3/L2 vertebra at ∼70° to be parallel to the endplates using a high precision diamond cutting blade (Accumtom-50, Struers). Slow feed speed (0.01mm/s) and blade rotation (1500 rpm) minimised any potential damage to the sample. Posterior elements of both vertebrae were removed from the spine segment using a scalpel. Angled cuts across vertebra and removal of posterior elements was required to achieve high resolution phase contrast tomography (further information is given in Supplementary Materials). Samples were then set in custom machined holders for use in the Deben CT5000 rig using epoxy resin and an alignment tool (Suppl. Fig. S1b).

### 2.2 Equipment and testing

Samples were held under cumulative compression from the bottom plate on the beamline with precise displacement control (300 nm resolution) using Deben CT5000 (Deben, UK) rig. A 500 N load cell was fitted to provide force readout. A 1 N preload was applied to secure the sample in place, and displacement held for a stress relaxation period of ∼15 min or until the force readout had settled. The first scan was then acquired and then a compression of 0.02 mm (∼2% strain / ∼1N peak load which is equivalent to a third of the animal's body weight) applied and held during the second ∼15 min long stress relaxation period and scan. This process was repeated a further three times to have a total of 8% applied strain for the last scan (Suppl. Fig. S3). A slow strain rate of 0.00167 strain/s (0.1 mm/min) was used to minimise the peak stress response and reduce stress relaxation time. Samples were enclosed in a humid environment which stopped them from dehydrating during testing and imaging.

### 2.3 In-line phase contrast synchrotron tomography

A synchrotron source was used for high signal-to-noise in-line phase contrast imaging of low abosprtion-contrast native soft tissue and for relatively short scans (12 minutes compared to hours for laboratory µCT) [23,25]. The Diamond-Manchester Imaging Branchline I13-2 [27] at Diamond Light Source, UK, is a partially-coherent x-ray source suitable for phase contrast imaging to resolve soft tissue structures without the use of stains. A pink beam (8–30 keV), generated by the minimum undulator gap of 5 mm, provided high flux to minimise scan time. The beam was filtered (with 1.34 mm pyrolytic graphite, 3.2 mm aluminium, and 0.14 mm iron, giving 27.6 keV weighted mean photon energy) to suppress lower energy photons and thus reduce beam damage. To minimise sample radiation dose and reduce ring artefacts by minimising scintillator defect brightness, shutters were used to reduce the beam size to be slightly larger than the field of view. Images were collected by a pco.edge 5.5 (PCO AG, Germany) detector (sCMOS sensor of 2560 × 2160 pixels) mounted on a visible light microscope of variable magnification. Magnification was controlled via rotation of a turret incorporating various scintillator-coupled objective lenses. A 2 × objective, coupled to a 500μm LuAG:Ce scintillator, gave a field of view of 4.2 × 3.5 mm and an effective pixel size of 1.625 μm. Exposure time per image was set to 0.15 s to achieve ∼75% saturation in flat-field images. A total of 5001 projection images were recorded for each scan over 180° of continuous rotation (‘fly scan’). Propagation distance (sample to scintillator) was set to 350 mm which provided sufficient in-line phase contrast to resolve IVD microstructure. With propagation after the sample, wave front distortion gives rise to Fresnel inference fringes which increase structure edge visualisation. The optimal propagation distance is proportional to the size of the structures being resolved and their composition, and therefore should be set empirically for heterogenous structures [23]. In this case, the propagation distance was incrementally increased until inner lamellae bundles were resolved. Propagation distance was chosen based on a set of scans on a test sample at 50 mm increments. Forty flatfield images were taken before each compression sequence with the Deben rig (without sample) in the beam path. Soft tissues exhibit stress relaxation when held under load, and so a stress relaxation period (∼15 minutes) and optimised short scans were needed to limit sample movement during scans.

### 2.4 Reconstruction and image processing

Reconstructions were generated with the open source, modular pipeline Savu [28]. Prior to filtered back projection, images were first normalised via flat- and dark-field correction, followed by corrections for radial distortions of the scintillator-coupled microscope [29] and ring artefacts. Paganin-based phase retrieval reconstructions [30], tailored to different analyses, were generated (Suppl. Fig. S4). High-level phase retrieval (δ/β=100) was used to enhance contrast and smooth lamellae structure for visualisation purposes. Low-level phase retrieval (δ/β=20), found to provide optimal contrast for fibre bundle feature size (Suppl. Fig. S4), was used for fibre tracing. Reconstruction without phase retrieval was used for DVC to preserve the micron-scale texture required for accurate tracking.

Image volumes were down-sampled to 16-bit for subsequent analyses. Avizo 2019 (Thermo Fisher Scientific, Waltham, Massachusetts, U.S.) was used for image processing and visualisation. Watershed segmentation for the rendering in Fig. 1a was required for the endplate due to bright edges from phase contrast.

### 2.5 Fibre tracing and point cloud seeding

Avizo 2019 XFiber Extension [31,32] was used for bundle tracing (Suppl. Fig. S5). ‘Cylinder correlation’ (length 40 px, angular sampling 5°, mask 5 px, outer 3 px) was used to enhance fibre-like structures. Region-of-interest edge artefacts were deleted by erosion (8 px). The erosion step was necessary due to a peculiarity whereby the cylinder correlates with the edge of the cropped region-of-interest and hence erosion by slightly more than the cylinder template is necessary. A threshold in the ‘Trace correlation lines’ module was applied to only select features which are fibre-shaped, tracing fibres with a high confidence level (minimum start 100, minimum continuation 80; Suppl. Fig. S5c–e). Fibres were sorted into lamellae using the labelling tool in the Avizo Filament workspace and exported as an xls file.

Matlab R2019a (MathWorks, Massachusetts, US) was used for point cloud seeding and post DVC processing. All Matlab functions were executed in batches asynchronously on a parallel pool to minimise computation time. A Matlab function was written to create point clouds which seeded evenly spaced points along the traced fibres (Suppl. Fig. S6). After the spreadsheet files are imported, the algorithm marches along the fibre calculating direction and distance between neighbouring points. Point clouds are created with fibre (*l f p n x y z)* information. Where, *l* is lamella number (increasing from outer to inner), *f* fibre number, *p* point number in that fibre, *n* point cloud number and *x y z* coordinate positions. Coordinate system is defined as *x* and *y* position in reference to IVD transverse plane and *z* giving IVD height position.

### 2.6 Digital volume correlation

Image volumes (16-bit) reconstructed without Paganin phase retrieval were used for DVC [22,33]. In DVC, each point represents the centre of a sub-volume whose displacement is tracked by correlating local sub-volume images from one load step to the next. Source code was supported by CCPi (Collaborative Computational Project in Tomographic Imaging). Sphere sub-volumes (diameter 30 voxels / 48 µm) centred at each fibre point in the reference image volume were correlated with the subsequent image volume (Suppl. Fig. S7). Choice of a smaller sub-volume allows high precision tracking but this often results in a trade-off with measurement uncertainty [22]. Sub-volume size was selected based on test runs (Suppl. Fig. S7). Reliable sub-voxel tracking was made possible by sub-voxel tricubic interpolation of 10k points randomly seeded which gave an overall <0.2 voxel (325 nm) accuracy. Displacement was found by nonlinear least squares optimisation of normalised sum of squared difference function.

Local minimum solutions were identified as a potential problem of fibre tracking, where correlation could be to a neighbouring fibre. DVC variables were chosen with this in mind: (i) PC-sCT reconstruction without phase retrieval was used for maximum fibre image texture, (ii) a dense point cloud (point every 8 µm along each fibre) with initial starting point specification, sorting of point processing order, and transfer forward of neighbouring point information, (iii) a high density (10,000) of sampling points within spherical sub-volumes to retain detailed image texture information whilst minimising cross-talk between fibres, (iv) and a fast nonlinear least squares optimiser coupled with a high-quality volumetric cubic-order interpolator. Post DVC, a space curve fitting process of third order polynomials weighted by DVC residual^−1^ was also used to moderate potential local minimum solutions and reduce the influence of displacement noise on calculated measurements (Suppl. Fig. S8).

To check tracking accuracy and robustness, a set of image volumes (using the first scan as a base) with different levels of added noise were correlated. Maximum and median values of noise (standard deviation of grey values in air) were measured in the scans added to the first scan. Suppl. Fig. S9 shows increasing strain uncertainty with increasing image noise. Strain uncertainty (standard deviation) was less than 0.0006 for images with the worst-case noise.

### 2.7 Parametric space curve fitting

Matlab R2019a (MathWorks, Massachusetts, US) function was written to fit third order polynomials to each fibre. Linear distance along fibre (*s*) was fitted against coordinate position (*x, y, z*) separately to give a set of 3 parametric space curve equations for each fibre.

(1) $$f_{x}\left(s\right)=p_{x1}s^{3}+p_{x2}s^{2}\;+p_{x3}s+p_{x4}$$

(2) $$f_{y}\left(s\right)=p_{y1}s^{3}+p_{y2}s^{2}\;+p_{y3}s+p_{y4}$$

( 3) $$f_{z}\left(s\right)=p_{z1}s^{3}+p_{z2}s^{2}\;+p_{z3}s+p_{z4}$$

Fits were weighted by DVC residual to reduce sensitivity to tracking noise. Curve fits were stored and output with distance along the fibre (*s*). Goodness of fit was evaluated using R^2^ and curve residual values (Suppl. Fig. S8). These equations were used in the fibre metrics calculations.

### 2.8 Fibre metrics calculations

Matlab R2019a (MathWorks, Massachusetts, US) functions were written and used to calculate all fibre metrics. Tangent vector (***t***), curvature (*k*), orientation (θ), displacement gradient in the fibre direction (***m***) and strain (L) was found for each point.

Tangent vector (***t***) to the fibre at each point was found by taking the derivative of the space curves Eqs. (1)-(3).

The explicit expression of curvature was taken from the Frenet-Serret equations [34] where orthogonal tangent (T) and normal (N) and binormal (B) vectors of a curve are given as

(4) $${\left[\begin{array}{c} T\\ N\\ B \end{array}\right]}^{\prime }=\left[\begin{array}{ccc} 0 & k & 0\\ -k & 0 & \tau \\ 0 & -\tau & 0 \end{array}\right]\left[\begin{array}{c} T\\ N\\ B \end{array}\right]$$

Where *k* is curvature and *τ* is the torsion of the curve. Curvature (*k*) can be calculated as

(5) $$k=\frac{∥f^{\prime }\left(s\right)\times f^{\prime \prime }\left(s\right)∥}{∥f^{\prime }{\left(s\right)}^{3}∥}$$

Where $f^{\prime }\left(s\right)$ and $f^{\prime \prime }\left(s\right)$ are first and second derivative of the parametric space curves respectively. Explicitly,

(6) $$k=\frac{\sqrt{{\left(f_{z}^{\prime \prime }{f_{y}}^{\prime }-f_{y}^{\prime \prime }{f_{z}}^{\prime }\right)}^{2}+{\left(f_{x}^{\prime \prime }f_{z}^{\prime }-f_{z}^{\prime \prime }{f_{x}}^{\prime }\right)}^{2}+{\left(f_{y}^{\prime \prime }f_{x}^{\prime }-f_{x}^{\prime \prime }f_{y}^{\prime }\right)}^{2}}}{{\left(f_{x}^{\prime 2}+f_{y}^{\prime 2}+f_{z}^{\prime 2}\right)}^{\frac{3}{2}}}$$

Fibre orientation was calculated using tangent vector information (***t***). Theta was defined as the angle in the lamella plane and thus given by,

(7) $$\tan\theta =\frac{\left|t\times a\right|}{t\cdot a}$$

Where a is the reference to the lamella plane, **a** = **t**{z=0}.

Displacement gradient (*u v w* in *x y z* directions respectively) from DVC was found in the fibre direction using the dot product with the fibre tangent.

(8) $$\begin{array}{c} m=▵\left\{\begin{array}{c} u\\ v\\ w \end{array}\right\}\cdot t \end{array}$$

This entailed a third order polynomial fit of displacements along the fibre with respect to distance along the fibre, *s* (Suppl. Fig. S10). The fitting process, weighted by DVC tracking residual, provided some filtering of the displacement values (Suppl. Fig. S10). High R^2^ (over 77% of the fibres had R^2^ > 0.8) and low residual for displacement fitting indicated reliable tracking. Lagrange strain was calculated from the displacement gradient along the fibre.

(9) $$L=m+\frac{m^{2}}{2}$$

### 2.9 Availability of DVC and analysis code

Matlab functions have been made available on Matlab Central File Exchange. DVC code is available from https://tomographicimaging.github.io/iDVC/.

### 2.10 Statistical methods

**Fig. 2:**
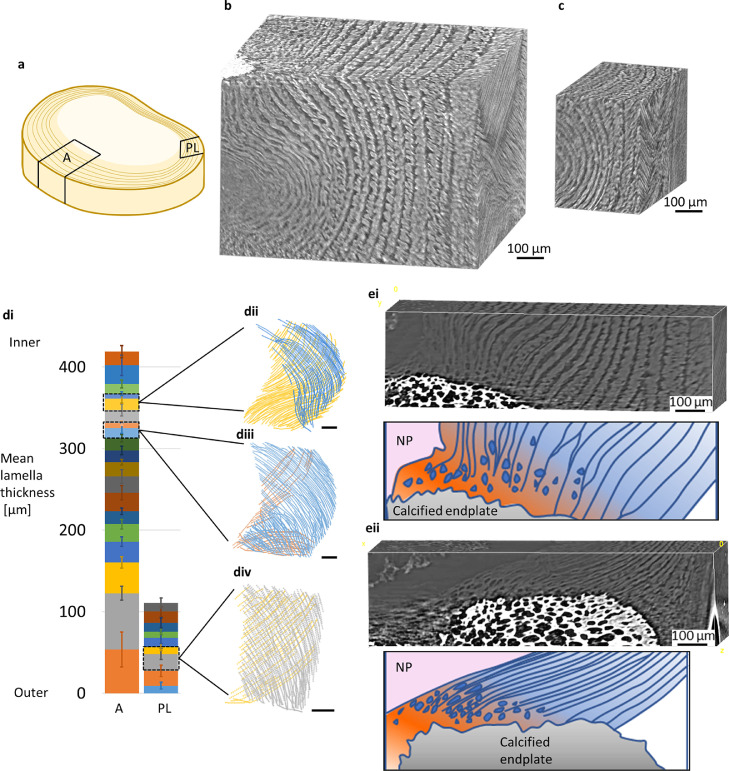
**Anterior and posterior-lateral regions have distinctively different annulus fibrosus structure. (a)** Location of anterior (A) and posterior-lateral (PL) regions. (**b)** Anterior volume render. (**c)** Posterior-lateral volume render. AF height is greatest in the anterior portion. Greater variation in lamellae morphology such as curvature and thickness is visible. (**d)** Mean lamella thickness for anterior and posterior-lateral regions. Overall, the anterior (A) has greater AF thickness and is composed of more lamellae in comparison to the posterior-lateral (PL). Anterior lamellae have a higher variation in thickness with lamellae generally decreasing from outer AF to inner AF, when compared to a more consistent lamella thickness in the posterior-lateral region. Partial or incomplete lamellae appear in both anterior and posterior-lateral regions (dii-iv; scale bars=100 μm). (**e)** AF-endplate attachment for (i) anterior and (ii) posterior-lateral regions. Attachment of lamellae (blue) to the cartilaginous endplate (orange with blue cells). (i) Anterior lamellae attachment is nearly normal to the endplate and inner lamellae are more loosely aligned when compared to (ii) posterior-lateral attachment which is at a shallower angle with tightly packed aligned lamellae. (For interpretation of the references to color in this figure legend, the reader is referred to the web version of this article.)

**Fig. 3:**
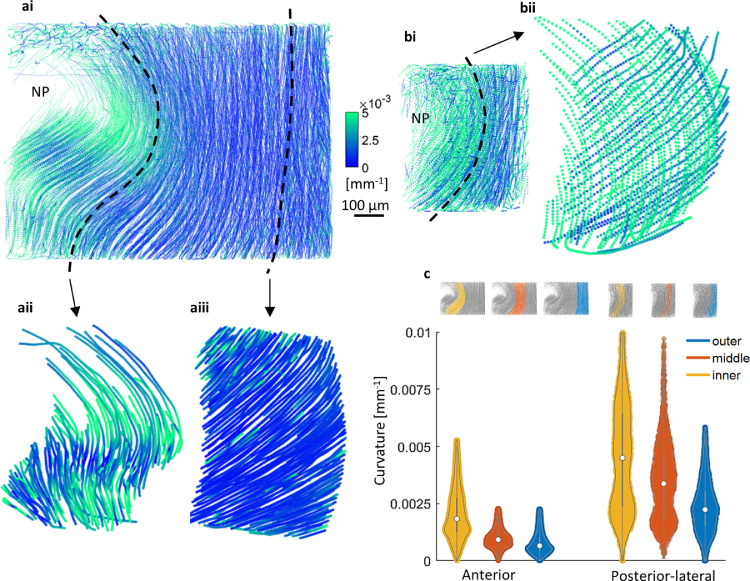
**Fibre curvature is determined by whole disc morphology, local AF structure and fibre orientation. (ai)** Anterior fibre curvature increased with AF depth. Inner lamella fibres had some local heterogeneity. (**aii)** example of inner AF fibre curvature. Curvature variation observed within a single lamella. Higher curvature was present at mid-height at an inflexion. (**aiii)** Nearly straight outer AF fibres. (**bi)** Overall fibre curvature increased with AF depth. (**bii)** Within a single lamella there was little variation, however an alternating pattern of low and high curvature between consecutive lamellae was observed due to the double curvature and asymmetric orientation of fibres in the posterior-lateral AF (Fig. 4). Steeper fibres had higher curvature (green in **bii**) which means that vertical curvature (bulge of the disc) was higher than the lateral curvature (around the circumference of the disc). (**c)** Distribution plots for curvature measurements for anterior and posterior-lateral regions are significantly different (*p* < 0.05). Posterior-lateral fibres overall had higher curvature than anterior fibres. Both regions showed a gradual increase in fibre curvature with AF depth. Plot distribution indicates local characteristics of fibre curvature – inner anterior increased variation relates to heterogeneity - and – posterior-lateral inner and middle bimodal distributions representing fibre-orientation-dependant results.

**Fig. 4:**
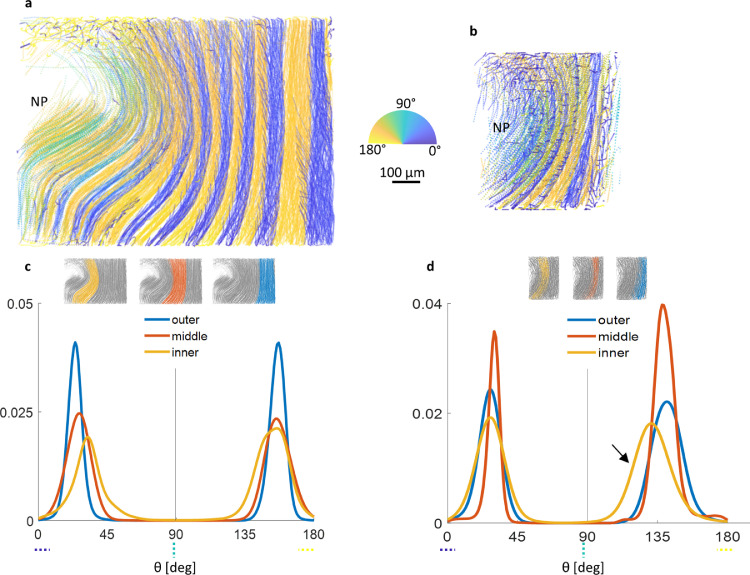
**Fibre orientation [deg] varies regionally and does not always follow a lateral inversion pattern for adjacent lamellae.** (**a)** Alternating fibre orientation in the anterior region. Fibre orientation steepens (towards 90°) with AF depth. No notable variation observed within a single lamella for the anterior region. (**b)** Alternating orientation of fibres in the posterior-lateral region. Fibres in the >90° group steepen (yellow towards green) with AF depth whereas the <90° (blue) group appears to remain similar. (**c)** Probability distribution of anterior fibre orientation showed a shift towards more vertical orientation (peaks converge) with AF depth. The distribution is symmetrical for all depths i.e. orientation between consecutive lamella is a lateral inversion. (**d)** Overall posterior-lateral fibres were steeper when compared to anterior. The asymmetric distribution means that the fibres were orientated in a skewed lateral inversion i.e. one group is more vertical than the other as shown by arrow. The more vertical fibre group (shown by the arrow) also had a wider distribution indicating some variation within these lamellae. Distribution peaks converge with AF depth with a similar magnitude to anterior (Suppl. Fig. S12). (For interpretation of the references to color in this figure legend, the reader is referred to the web version of this article.)

**Fig. 5:**
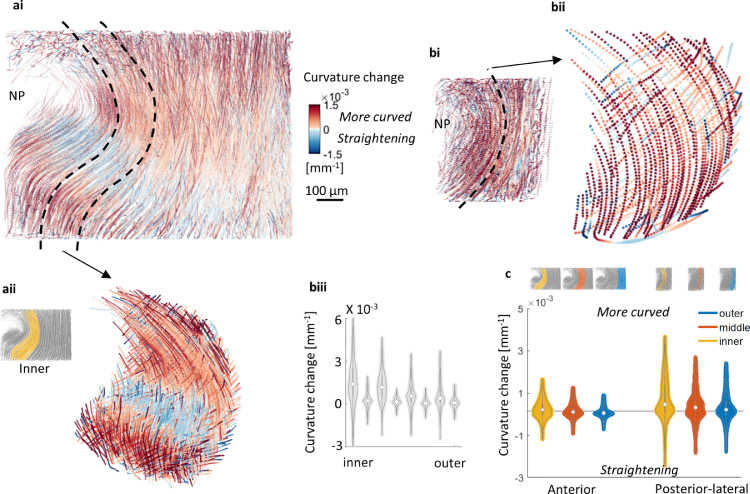
**Change in fibre curvature is linked to tissue morphology and fibre orientation. (ai)** Magnitude of anterior fibre curvature change increases with AF depth with little change displayed in outer AF. (**aii)** Fibre curvature change for inner AF. A group of fibres show an increase in curvature (red) whereas others straighten (blue) suggesting tissue morphology changes. (**bi)** Overall a higher change in curvature was found throughout the posterior-lateral region. (**bii)** Displaying two consecutive lamellae shows an alternating pattern of higher increase in curvature and lower increase in curvature or straightening. Changes in curvature were higher for more vertically orientated fibres i.e. curvature changes were more dominant in vertical direction (bulging of the disc). (**biii)** Change in curvature plotted for individual lamellae showing alternating pattern (**c)** Regional comparison of change in fibre curvature. Anterior fibre curvature change has a gradual increase with depth. Variation in anterior region is due to localised tissue morphology changes, mostly found in inner lamellae. Variation in the posterior-lateral region is due to whole AF deformation and fibre orientation. Inner-middle posterior-lateral fibres had the most prominent pattern in alternating high and lower increase in curvature as indicated by a bimodal distribution. Notably, higher curvature changes were present in all posterior-lateral groups when compared to anterior groups. (For interpretation of the references to color in this figure legend, the reader is referred to the web version of this article.)

**Fig. 6:**
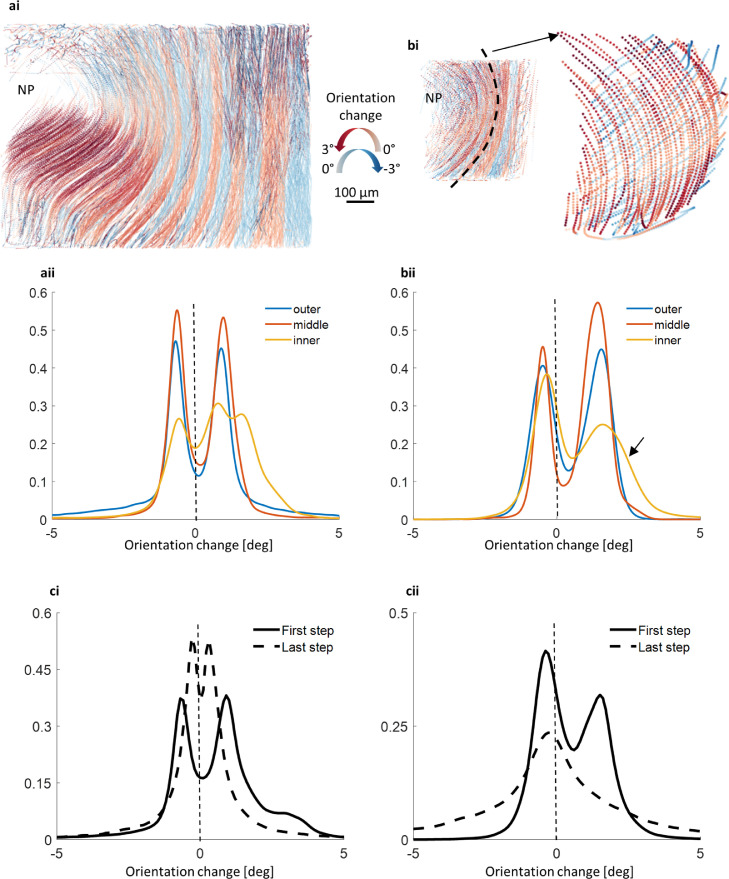
**Change in fibre orientation [deg] with load is non-linear and region specific. (a)** Change in orientation of anterior fibres after the first load (2% compression) (**ai)** Recruitment of fibres to direction of loading. Fibres changed orientation towards the transverse plane creating an alternating pattern of clockwise and anticlockwise reorientation for consecutive lamella. Change in fibre orientation increased with AF depth. (**aii**) Change in orientation probability distribution for anterior regions. A symmetrical distribution centred around 0°-outer & middle groups-indicates equal reorientation for consecutive lamellae. Inner region has some measurements with higher reorientation (found at mid height in ai)). (**b)** Change in orientation of posterior-lateral fibres after the first load. (**bi)** Similar pattern of alternating reorientation for consecutive lamella. (**bii)** Separation of peaks was greater for posterior-lateral fibres and therefore higher reorientation overall when compared to anterior. Distribution is not centred around 0° particularly for inner lamellae fibres. Inner fibres show asymmetrical distribution - steeper orientation fibre group (Fig. 4d) with greater reorientation – and a broader distribution (arrow) showing local variation within inner lamellae (higher at top and bottom of lamella as shown in bi)). (**ci)** Change in orientation of anterior fibres at the first load increment (preload-2% compression) and at the last load increment (4–6% compression). Peaks converge showing a decrease in fibre recruitment with load. (**cii)** Change in orientation of posterior-lateral fibres at the first load increment (preload-2% compression) and at the last load increment (4–6% compression). Fibre recruitment is initially higher than anterior fibres but converges to zero for higher loads. Change in orientation measurements (c) for anterior and posterior-lateral regions are significantly different (*p* < 0.05).

Mean lamella thickness and standard deviation (error bars) in Fig. 2d was calculated from nodes of triangulated lamellae surfaces. KS test was used to show significant differences (p<0.05) between all groups in distribution plots in Figs. 3c, 4c, 4d, 5c, 6aii, 6bii, 6c, and 7c. Three samples were tested in total. The following figures display data from one sample. Results from the two other samples can be found in Supplementary Materials.

**Fig. 7:**
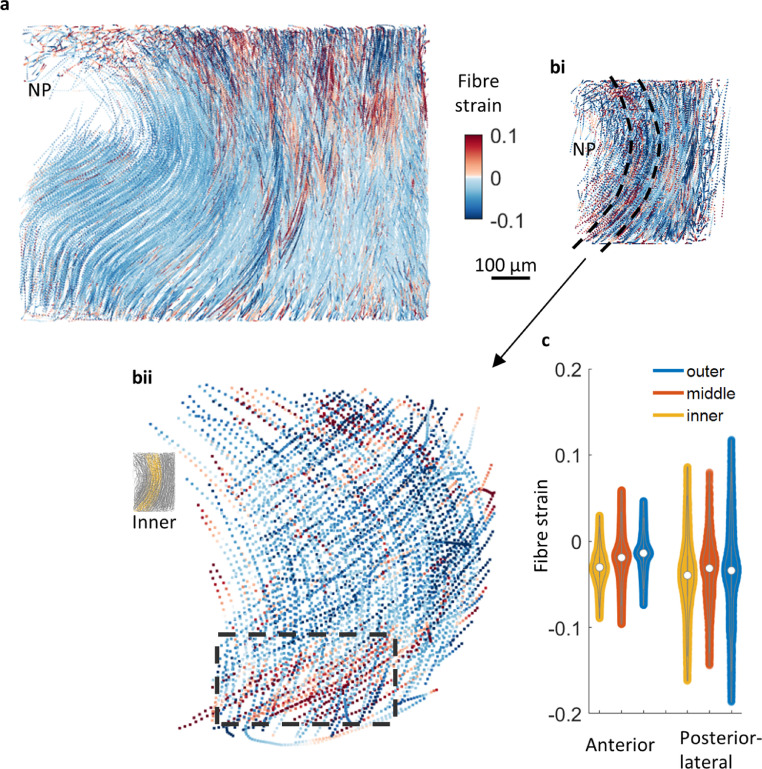
**Fibre strain (a)** Anterior fibre strains are overall compressive (shortening from reference state) with some local variation of higher compression and tension toward the superior vertebral body. (**bi)** Posterior-lateral fibre strain magnitudes are generally higher when compared to anterior measurements. (**bii)** Inner posterior-lateral fibres show a general trend toward compressive strain with tensile strain observed near the endplate junction (box) (**c)** Comparison of fibre strain between regions. Overall, there is a balance between tensile and compressive strain (lengthening and shortening) with anterior fibre strains collectively lower in magnitude than in the posterior-lateral region.

## 3 Results

### 3.1 Optimised sCT imaging of whole intervertebral disc reveals distinct regional collagen bundle architecture

#### 3.1.1 Imaging collagen bundle architecture from a whole native intervertebral disc

The entire AF collagen bundle architecture of an intact rat lumbar IVD was resolved using phase contrast sCT (Fig. 1). A relatively large field-of-view (4.2 × 3.5 mm) allowed high resolution tomography of intact IVD where the smallest resolved feature was collagen fibre bundle width (∼5 µm) (Fig. 1e, Suppl. Video 1). A good contrast-to-noise ratio (Suppl. Fig. S2, Suppl. Table 1) with phase retrieval reconstruction meant that fibrous structures could be successfully traced resulting in >10k fibres typically identified per region. Since lamellae could be identified across the full AF thickness (Fig. 1d), fibres could be labelled by each lamella (Fig. 1f). Lamella labelling enabled subsets of data such as individual lamellae and sub-region (inner, middle and outer) to be used in comparisons between different IVD regions.

#### 3.1.2 Conspicuously different regional annulus fibrosus structure

The posterior-lateral and the anterior AF regions were taken from each scan for comparison (Fig. 2a). The anterior region is overall larger in height and AF thickness than the posterior-lateral region (Fig. 2b and c). Mean lamella thickness tends to decrease from outer to inner lamellae for the anterior region when compared to the posterior-lateral region which has fewer, thinner but more consistent thickness of lamellae (Fig. 2di). Interestingly, incomplete lamellae can be found in both regions (Fig. 2dii–iv).

Careful sample placement by aligning the sample endplates to be parallel with the beam meant that it was possible to visualise the previously unresolved by sCT AF-cartilage endplate attachment which is susceptible to damage, particularly for inner posterior-lateral lamellae attachment. Fibre bundles changing orientation to meet the endplate and individual chondrocyte-like cells were resolved signifying the cartilage endplate (Fig. 2e, Suppl. Video 2). The anterior AF attachment appears to be more normal to the endplate (Fig. 2ei) than compared to the oblique attachment of posterior-lateral AF (Fig. 2eii). Anterior attachment also appears to be less formally structured than the highly aligned lamellae in the posterior-lateral attachment.

#### 3.1.3 Structure across hierarchical scales influences regional differences in fibre curvature and orientation

Curvature and orientation were calculated locally (spatially, every 8 µm) along each individual fibre. Over 200,000 measurement points for 10,000s fibres in each region gave an incredibly rich dataset to be studied. Our approach used a hierarchical point cloud so that each point was assigned a fibre number and lamellae number (Fig. 1f, Suppl. Video 5). This meant that the measurements could be interpreted regionally and across hierarchical scales. Lamellae were divided into inner, middle and outer regions for both the anterior and posterior-lateral volumes for comparison purposes to observe strain transfer from NP pressure to outer lamellae and identify local differences.

Curvature of a fibre was calculated at each point from its space curve using Frenet-Serret equations [34], measuring curvature at microscale spatial resolution in three-dimensions giving a complete description of individual fibre morphology within a large region of an intact disc (Fig. 3). This was performed as it is known that curvature of fibres significantly influences tissue mechanical properties. In this case, fibres with local changes in curvature along their length give the tissue higher compliance and lower load transfer capacity, when compared to a consistent curvature along their length. Overall, the posterior-lateral region had higher curvature (Fig. 3c). The curvature measurements for the posterior-lateral region displayed an alternating pattern between adjacent lamellae (Fig. 3bii). Two peaks in Fig. 3c (posterior-lateral, middle and inner) represent the two groups in the alternating pattern of fibre curvature which is a combined result of fibre orientation and double curvature geometry in the posterior-lateral region of the disc: fibres orientated with a steeper incline have a higher curvature (green in bii). The anterior region had overall lower curvature values with outer fibres being nearly straight (Fig. 3aiii) and local variations with some regions of higher curvature for inner fibres (Fig. 3aii).

It is important to characterise fibre orientation since they are load bearing along their axis and therefore their orientation determines the anisotropic (or directional) mechanical properties of the tissue. Furthermore, fibre reorientation or recruitment with load is related to soft tissue non-linear behaviour giving compliance at low loads as fibres orientate to the direction of loading and high strength at high loads upon recruitment. Orientation every 8 µm along each fibre was measured where theta is the angle from the transverse plane: 0^o^ and 180^o^ are horizontal orientations, 90^o^ is vertical (Fig. 4). Fibre orientation in posterior-lateral region was more vertical when compared with anterior fibres. Modal values from the orientation probability distributions show fibre pitch (angle to vertical) difference of ∼11° between the two regions (Suppl. Fig. S11, Repeats shown in Suppl. Fig. S12). In both regions, fibres transition by 6° towards the vertical axis with radial depth from outer lamellae towards inner lamellae (Fig. 4c and d, Suppl. Fig. S12). As might be expected, a symmetrical distribution centred around 90^o^ was found for anterior fibre orientation, indicating a lateral inversion (or mirrored) pattern for fibre orientation in adjacent lamellae (Fig. 4c). The plot for orientation of posterior-lateral fibres, however, had an asymmetric distribution meaning that the fibres followed a skewed lateral inversion pattern where orientation alternates in pitch for adjacent lamellae (Fig. 4d). Notably, there was no local variation within a single lamella but with the exception of the more vertically orientated group of fibres in the posterior-lateral region.

### 3.2 Load-induced changes to fibre curvature, orientation and strain reveals regional mechanical tissue function

#### 3.2.1 Displacement of individual fibres can be tracked at nanometre resolution using digital volume correlation

Signal- and contrast-to-noise ratios remained stable for all scans during the compression sequence (Suppl. Table 1) enabling nanometre displacement tracking using DVC. Sub-voxel tracking gave <0.2 voxel (325 nm) accuracy overall. Discrete fibre displacement tracking was possible using fibre point clouds for DVC (Fig. 1f, Suppl. Video 5). Crucially, this meant that a direct link between fibre organisation and morphology, their changes with load and fibre strain could be quantified and visualised, in comparison with typical continuum strain measurement which provides information on averaged local tissue mechanics.

Quantities such as curvature and strain were derived from the fitted curves at the displacement measurement locations as opposed to direct use of the displacement values themselves. Space curve fitting confidence levels of 97% fibres with R^2^ >0.9 and mean residual <0.01 voxel across all compression steps strongly indicated that individual fibres were being tracked (Suppl. Fig. S8), as does simple observation of dynamic loading sequences (Suppl. Video 5).

#### 3.2.2 Regional differences in change of fibre curvature and orientation as morphological indicators of tissue compliance

After sample loading, fibres were seen to both increase in curvature (red) and straighten (blue) (Fig. 5a), with differences related to anatomic region, lamellae position across the AF, and location within the lamellae. The posterior-lateral region exhibited an alternating pattern of higher and lower increase in curvature, particularly within mid-inner regions (Fig. 5b). Lamellae with more vertically oriented fibres showed greater curvature changes than lamellae with less vertically oriented fibres. Curvature changes were greater overall and more varied in the posterior-lateral region when compared to the anterior region (Fig. 5biii and c). Localised regions of fibre straightening were found in the anterior region signifying tissue morphology changes, particularly for mid-height inner fibres where there was a slight inflexion (Fig. 5 aii). Change in curvature remained a constant magnitude with increasing load for the anterior region whereas magnitude increased substantially for posterior-lateral (Suppl. Fig. S13). These results are consistent with predictions which can be made from the structural characterisation above; a compliant inner anterior region inferred by small, localised curvature changes accommodating for tissue morphology changes of an intrinsic inflexion, and a less compliant posterior-lateral region inferred by consistent curvature throughout individual lamella whose change with load implies radial bulging of the entire AF region.

Fibres were recruited upon loading as they re-orientated towards the transverse plane (Fig. 6). Anterior fibres re-orientated in a symmetrical fashion with fibres from neighbouring lamellae re-orienting equally in a clockwise and anticlockwise rotation (Fig. 6a). In general, fibres in outer lamellae had consistent reorientation when compared to fibres within inner lamellae which had more variation in reorientation (variation shown as broader peaks in Fig. 6aii and 6bii). This variation can be seen as a subtle change from higher reorientation at top and bottom when compared to mid-height (Fig. 6bi). Posterior-lateral fibres, however, did not re-orientate equally in a clockwise-anticlockwise rotation for neighbouring lamellae. Instead, posterior-lateral fibres had an asymmetric distribution of fibre reorientation (Fig. 6bii) which when plotted spatially showed alternating reorientation magnitude between higher and lower for neighbouring lamellae. Fibres which initially were steeper had the highest change in orientation anticlockwise towards the transverse plane (Fig. 6b). The overall amount of re-orientation decreased with increasing load (Fig. 6c, repeat in Suppl. Fig. S14). Decreasing re-orientation with load was more evident in the posterior-lateral region-of-interest.

#### 3.2.3 Transfer of strain to fibre-level is highly localised

Overall, applied compressive loading generated higher strain magnitudes in the posterior-lateral region than the anterior region (Fig. 7). Average strain magnitude increases with radial depth for the anterior region (Fig. 7a and c) but remains relatively constant for the posterior-lateral region (Fig. 7b and c). A region of tensile strain was obeserved near the endplate for inner posterior-lateral fibres (Fig. 7 bii) where the fibres connect at an oblique angle. When measured incrementally, fibre strain increases with applied compressive load particularly in the posterior-lateral region (Suppl. Fig. S15).

The strain measurements should be considered concurrently with other tissue deformation mechanisms such as the change in fibre curvature and orientation measurements shown above. The anterior region exhibited greater changes in tissue morphology for example the straightening of a slight inflexion of inner lamellae from NP pressure when the disc is loaded and microstructural reorganisation such as continued fibre reorientation at higher loads (Fig. 6ci). Higher strain values were observed in the inner lamellae where curvature change variation and orientation changes were greatest.

Posterior-lateral tissue morphology is likely to be of low compliance due to inherent high tissue curvature that increases with load. Fibres have an initial high reorientation with load, but reorientation is not linear as it is not present for increasing loads (Fig. 6cii). The combination of high tissue curvature, limited fibre reorientation and relatively consistent higher strain values throughout the posterior-lateral AF indicated lower tissue compliance and increased load bearing when compared to anterior AF.

## 4 Discussion

This is the first demonstration of tracking discrete fibres in 3D within an intact IVD. AF displacement or fibre strain has previously been inferred from a few point measurements using microscopy [12] or markers and stains [16,[35], [36], [37]]. These techniques require dissection which releases residual strain and alters tissue mechanical behaviour. In this work, approximately 200k points were evaluated within each AF region, spaced every 8 μm along 10k individual fibres (Fig. 1). Here, IVD fibre architecture under load was observed and quantified by tracking PC-sCT of native intact IVD without the use of stains. A microstructure linked DVC approach, with flexibility to specify the point cloud, made it possible to track individual fibres. This provides a more mechanistic study of soft tissue mechanics than previous continuum measurements [26] making it possible to link microstructural characterisation such as fibre curvature and orientation to localised load-induced changes with fibre strain measurement. Enabled by an unmatched combination of a large field-of-view and high resolution offered by sCT, this analysis spanned across multiple length-scales comparing microstructural measurements between AF regions, across the full AF depth, between individual lamellae and even along individual collagen fibre bundles. The cell-scale fibre measurements presented here have relevance to understanding disc pathology as disease progression is related to cell mechanosensing in a changing fibrous environment [10]. Soft tissues and their constituents (collagen, elastin, proteoglycans) have strongly nonlinear mechanical responses related to their microstructure, contributing to the complex and local variation in mechanical environment of residual strain in unloaded IVD and mechanically loaded IVD. This greatly increases the importance of preserving tissue structure and localised residual strain patterns as even a seemingly homogeneous tissue will behave in an inhomogeneous manner [11].

The AF lamellar structure is not particularly well detailed in the literature, widely being described as having concentric lamellae. Our results showed each lamella was not continuous around the whole disc and thickness varied with circumferential, radial and height position in the disc (Fig. 2). We observed anterior lamellar thickness decreased with AF depth whereas posterior-lateral thickness was relatively thinner and more consistent. To our knowledge, the only documentation of lamellar thickness measurements are from tissue sections which showed similar results for posterior-lateral lamellae thickness of consistent thinner lamellae but the opposite to our results of change in anterior lamellae thickness with AF depth [3]. Measuring directly from tissue sections not only releases residual strain, causing the tissue to distort from its native structure, but also simplifies a 3D curved structure into a 2D plane which is likely to give erroneous results. Our results have used the average thickness of each lamellae from a regional volume in an intact disc which importantly accounts for 3D structure.

Incomplete lamellae were found in both regions (Fig. 2d). It has previously been hypothesised that the number of incomplete lamellae varies circumferentially [38]. Our observations suggest that incomplete lamellae may be related to lamella thickness as they are found where lamellae thickness is at its finest (inner anterior and posterior-lateral). Consideration of lamellae thickness and incomplete lamellae have been generally neglected in modelling studies which describe AF lamellae as evenly spaced and continuous [17]. Simplifying models to answer a specific question or to reduce computational load may be the reason in some cases. However, our detailed characterisation of regional variation in AF structure will be useful as AF micromechanical models continue to move toward more realistic and detailed representations of fibre morphology [39].

The anterior and posterior-lateral regions were found to have distinctly different tissue morphologies. The anterior AF attachment was nearly normal to the endplate and lamellae arrangement was less aligned compared to the parallel alignment of the posterior-lateral lamellae which had an oblique attachment to the endplate (Fig. 2e). Resolving this structure at high resolution within an intact disc for the first time is important due to the high likelihood of damage, particularly near the inner lamellae [6] and due to its function in transferring load. The fibrocartilage structure of the uncalcified endplate was present in the inner AF for both regions which indicates a change in elastic modulus to help dissipate load at the soft-hard AF-endplate interface. Drawing from joint enthesis similarities and observed in our results, the quantity of uncalcified fibrocartilage correlates with the range of insertional angle change that occurs with load [40,41].

Fibre curvature patterns showed outer to middle anterior fibres to be nearly straight (Fig. 3aiii). Posterior-lateral fibres increased in curvature with AF depth without variation along their length (Fig. 3bii). Inner anterior fibres, however, had local variation in curvature along their length (Fig. 3aii) which means they have the flexibility to deform and straighten to a consistent curvature. The consistent high double curvature and aligned organisation of lamellae in the posterior-lateral region suggests lower remaining capacity for flexibility and possibly lower tissue compliance.

There is overall good agreement with previous studies showing regional variation in fibre orientation [2,19,20,42]; fibre orientation is more vertical in posterior regions. Posterior-lateral fibres had steeper (11° pitch difference) orientation when compared to anterior fibres (Fig. 4, Table 1, Suppl. Fig. S12). Circumferential variation in fibre orientation has been previously observed albeit our results had overall shallower fibre angle, particularly for posterior-lateral regions (Fig. 4, Table 1) [2,19,20,42]. However, fibre orientation variation with depth has been less well-documented. Our results showed a gradual steepening of fibres with increasing AF depth (Fig. 4c and d, Table 1, Suppl. Fig. 12). Discrepancies between previous findings and our results may be related to intact samples retaining their tensile residual strain contributing to overall shallower fibre angle and fibre orientation variation patterns echoing residual strain distributions [11,17].

**Table 1:** **Regional fibre orientation.** Peak values from probability distribution plots in Fig. 4c and d.

	Anterior [deg]		Posterior-lateral [deg]	
Outer AF	23.80	156.04	26.87	140.50
Middle AF	26.22	156.07	30.21	138.52
Inner AF	31.42	154.76	27.28	130.02

**Table 2:** Change in regional fibre orientation with 2% sample compression. Peak values from probability distribution plots in Fig. 6a and b.

	Anterior [deg]		Posterior-lateral [deg]	
Outer AF	-0.7	0.89	-0.5	1.55
Middle AF	-0.65	0.96	-0.49	1.42
Inner AF	-0.58	0.78	-0.35	1.59

Our results showed a lateral inversion pattern for fibre orientation in the anterior region which is widely accepted in the literature (sometimes described as a criss-cross pattern). The posterior-lateral region, however, had a skewed lateral inversion where one group of fibres was more vertical than the other (Fig. 4d) which had implications on how fibres changed in curvature and orientation under loading. Re-orientation of fibres is partly responsible for the higher flexibility of soft tissues at lower loads. Strain transfer through the tissue at low loads is dominated by re-orientation of fibres as they are recruited towards the direction of loading, compared to higher loads when fibres become increasingly strained. Global tissue response from mechanical testing [13,43] or lower resolution volumetric imaging such as MRI has already shown regional variation in disc mechanics [44,45]. How this regional variation is related to fibre architecture in an intact disc has not yet been characterised.

Results in Fig. 6a and b and Table 2 shows re-orientation of fibres towards the transverse plane with applied load. Reorientation of fibres in response to the applied load has been reported in dissected AF tissue under tensile load [12,46]. There are two differences to note between the results in Fig. 6 and previous studies. Firstly, our results overall had lower magnitude in fibre re-orientation when compared to previous studies. Secondly, previous findings showed a positive linear relationship between fibre re-orientation magnitude and applied load whereas Fig. 6 c shows a decrease in fibre reorientation magnitude with increasing load. Fibre re-orientation may have been influenced by the presence of other tissues, such as the pressurised NP and the attachment into the endplate, shown in our intact samples for the first time when compared to the dissected samples under direct tensile loading used in previous studies where endplates are removed. This demonstrates the advantages of testing intact samples to maintain natural boundary conditions and as a better representation of AF physiological loading including load transfer from pressurised NP. This is a significant finding as the magnitude of fibre re-orientation indicates a change of tissue function from a flexible compliant tissue to a stiffer load bearing tissue. Our results also show a more reduced re-orientation in posterior-lateral region at higher loads suggesting that this region is functionally less compliant and stiffer than the anterior region during disc compression. This would also increase the tissues susceptibility to damage as loads increase.

A positive relationship between initial fibre angle and amount of re-orientation has been shown in dissected tissue but without information on disc region [12]. Our results match this trend but valuably compare the initial fibre angle and effect on subsequent re-orientation with load across AF depth and between disc regions. Taking depth observations for the more robust anterior region for example, inner anterior fibres initially had steeper orientation and greater re-orientation with applied load than outer anterior fibres (Figs. 4c and 6a). This implies inner anterior lamellae are adapted to have more compliance than outer anterior lamellae. Comparing the effect of initial fibre orientation on re-orientation between regions was also possible. Posterior-lateral fibres initially had steeper fibre angle and higher re-orientation at lower loads but almost no fibre re-orientation at higher loads (Figs. 4d and 6cii) when compared to anterior region measurements. This may indicate a more rapidly increasing stress in the posterior-lateral region than the anterior region as fibre recruitment occurs more readily.

Mechanisms responsible for fibrous soft tissue non-linear mechanical response were quantified; at low loads, strain transfer was dominated by microstructural reorganisation such as fibres re-orientating and change in tissue curvature while at higher loads fibres became increasingly strained. An MRI *in situ* study showed radial displacements were significantly higher in anterior than posterior [47]. Higher resolution sCT imaging used here made it possible to directly quantify the microstructural morphology changes associated to their observations. As the disc was loaded and NP pressurised, the anterior tissue morphology had a slightly inflexion which meant that there were localised regions of inner anterior fibres which straightened whilst other localised regions became more curved when the disc was loaded (Fig. 5a). The posterior-lateral region however, had more uniform behaviour with a generalised increase in curvature (Fig. 5b). Fibre response within inner anterior lamellae suggests a transition zone between anterior AF and NP which acts structurally as a dampener facilitated by local variations in tissue morphology changes with disc loading. In comparison, the more formal organisation and consistent curvature of posterior-lateral lamellae does not allow for such local variations in morphology changes and is therefore less adaptable to load.

It is notable that interpretation of the fibre strain results should recognise that strain is measured with respect to an initial reference state that, in the IVD, is known to embody spatially varying patterns of residual strains [11,17]. The reference state for the measurements presented here is the mechanically unloaded intact disc maintained between adjacent endplates and therefore “preloaded” due to swelling pressures within the nucleus pulposus and within the annulus fibrosus itself. It is clear from available modelling and experimental evidence that some of these residual strains will be tensile, with associated fibre stretch. Measurement of compressive strain therefore does not imply compressive stress but perhaps shortening from an initially-stretched reference state. Fibre strain values varied somewhat through the disc. Variations in fibre strain with depth, observed in Fig. 7, can be linked to the microstructural changes measured here (fibre re-orientation and curvature). For example, tissue curvature changes and higher fibre recruitment for inner anterior regions meant that strain transfer was mainly within this inner tissue layer with a gradual fibre strain decrease towards outer layers (Fig. 7c). On the other hand, the less compliant tissue morphology of consistent curvature in the posterior-lateral region combined with eventual decreased fibre recruitment resulted in high strain transfer through to fibre level at higher loads (Fig. 7c). Furthermore, measurements showed high tensile fibre strain focussed at the posterior-lateral endplate junction (Fig. 7bii). This observation is important since strain localised at the endplate increases the risk of damage, supported by clinical findings that the endplate junction is a common site for herniation [6].

## 5 Future study and conclusions

With the highly localised fibre morphology patterns presented in this work, showing strong regional, lamellar, and even intra-fibre variations in orientation and curvature, it is likely the residual strain patterns are equally complex. The disc residual strain environment is not fully known or contributing factors understood. Characterisation has been limited by methodology, for example tracking deformations as residual strain was released by dissection is a surface measurement and does not include pressurisation from NP or inherent strains related to endplate attachment [11]. Modelling approaches have attempted to determine the residual strain as a result of swelling pressure and would benefit from the detailed structural characterisation in our results [17]. It is possible to obtain a definitive measurement of fibril pre-strain from collagen d-period using small angle x-ray scattering as shown in cartilage, but this has been limited to thin biopsy section samples or through-sample measurements [48].

There are limitations associated with using synchrotron source x-rays which were required for the high-resolution phase contrast of native tissue: Limited synchrotron availability hinders the time required to produce multiple replicates. Although the PC-sCT presented here has relatively large field-of-view when compared to laboratory microCT [25] and other microscopy techniques [49], limited beam size restricted this study to rat lumbar samples which do not truly represent the clinical case. It is not known whether rat lumbar IVDs are susceptible to damage in the same manner as human IVDs. However, rat lumbar IVDs have similar geometry [50], similar mechanical properties when normalised by their geometry [51], and quadrupedal animals have been reported to be under similar or higher levels of compression due to muscle and ligament forces when compared with human discs [52,53]. Although intact IVD was tested, it is important to note that there was a requirement for careful sample preparation and placement in the beam in order to achieve high resolution imaging, such as removing calcified material (posterior elements) from the beam path, which may affect overall spine mechanics. The *in situ* PC-sCT presented here was restricted to stepped loading rather than dynamic loading due to the scan time. It is known that dynamic, complex loads such as combined flexion and torsion, are more physiological and are more likely to cause tissue damage [54,55]. This study employed simple compressive loading but there is no barrier to other loading modes or tissues, for example torsion of spine segments, tensile loading for tendons and ligaments and pressurisation for vascular structures. Future studies may make use of further advances in PC-sCT or may focus on shorter imaging times enabling increasingly dynamic *in situ* imaging, albeit with a compromise in resolution. Time-resolved fibril strain can be measured using small angle x-ray scattering but strain is measured at a single point and averaged through the sample thickness [48,56].

The methodology described here is of broad relevance to the scientific and biomedical communities as it can be applied to any material system and volumetric imaging modality capable of resolving structural reinforcement fibres to a level where image-based fibre tracing is possible. Other imaging modalities such as confocal and multiphoton microscopy which can resolve individual fibres and have proven compatibility with *in situ* loading will also benefit from this measurement approach. The ability to perform detailed characterisation of numerous individual collagen fibres from *in situ* imaging of intact tissues and structures goes some way to enhance our fundamental understanding of tissue biomechanics and insight to discover structures which cause tissue regions to be more susceptible to damage. In addition to experimental studies, this approach generates rich and detailed information vital for future structural model development and validation.

This study provides insights into variations in IVD fibre architecture between anterior and posterior-lateral regions. Clinical observations show that damage most likely occurs in the posterior-lateral region of human disc [6,7] and that disease progression is related to cell mechanosensing in a changing fibrous environment [10]. There is also growing evidence of regional variation in disc fibre architecture [2,19,20,42], mechanical properties and residual strain [10,17,18], thus emphasising the importance and need for studying the IVD fibrous environment in intact samples as characterised here. Tissue morphology which has lower flexibility capacity and a reduced fibre recruitment at higher loads, as found in the posterior-lateral region, are indicative of decreased compliance, increased load transfer which leads to a higher overload susceptibility and eventual tissue damage.

## CRediT authorship contribution statement

**C.M. Disney:** Data curation, Formal analysis, Writing – original draft, Writing – review & editing. **J. Mo:** Data curation, Writing – review & editing. **A. Eckersley:** Data curation, Writing – review & editing. **A.J. Bodey:** Data curation, Writing – review & editing. **J.A. Hoyland:** Writing – review & editing. **M.J. Sherratt:** Writing – review & editing. **A.A. Pitsillides:** Writing – review & editing. **P.D. Lee:** Writing – review & editing. **B.K. Bay:** Data curation, Formal analysis, Writing – review & editing.

## Declaration of Competing Interest

The authors declare that they have no known competing financial interests or personal relationships that could have appeared to influence the work reported in this paper.

## Appendix Supplementary materials

Image, application 1

Suppl. Video 1 **Overview of anterior AF region of interest image volume and traced fibres**. Transverse slice of the disc is shown. The anterior region of interest is highlighted and render shown. Anterior render is clipped from the outer lamella to inner lamella showing collagen fibre bundles alternating orientation between adjacent lamellae. Upon reverse clipping, traced fibres are revealed with displayed colour map indicating alternating fibre bundle orientation. Rendered image volume is displayed over the traced fibres. Image, Video 1

Suppl. Video 2 **Endplate-disc boundary**. Sagittal slice of the sample is shown. A region at the endplate-disc boundary is selected and image volume render shown. White-grey indicates calcified endplate, orange-yellow shows cartilaginous endplate and AF attachment. Importantly structures at this boundary can be resolved with our improved imaging results. Image, Video 2

Suppl. Video 3 **Points exported from Avizo fibre tracing for anterior region of interest, grouped and labelled by lamellae**. Note that points are not regularly spaced along each fibre. Image, Video 3

Suppl. Video 4 **Lamellae labelled fibre point cloud for anterior region of interest**. Points were seeded at regularly spaced intervals (5 voxels/ 8 µm) along each fibre. Image, Video 4

Suppl. Video 5 **Digital volume correlation tracked displacement of the lamellae labelled point cloud for anterior region of interest. Measurement points were spaced every 8 µm along each fibre and tracked using digital volume correlation**. Compression (from the bottom) of the sample can be seen. This visualisation importantly shows that discrete fibres can be tracked using digital volume correlation. Image, Video 5
