# Supplementary material for: Regional variations in discrete collagen fibre mechanics within intact intervertebral disc resolved using synchrotron computed tomography and digital volume correlation
Source: Acta Biomater. 2022 Jan 15;138:361–74. doi: 10.1016/j.actbio.2021.10.012 (PMC8904373; doi:10.1016/j.actbio.2021.10.012)
Supplement: Supplementary file 1 [file mmc1.pdf]

Supplementary information

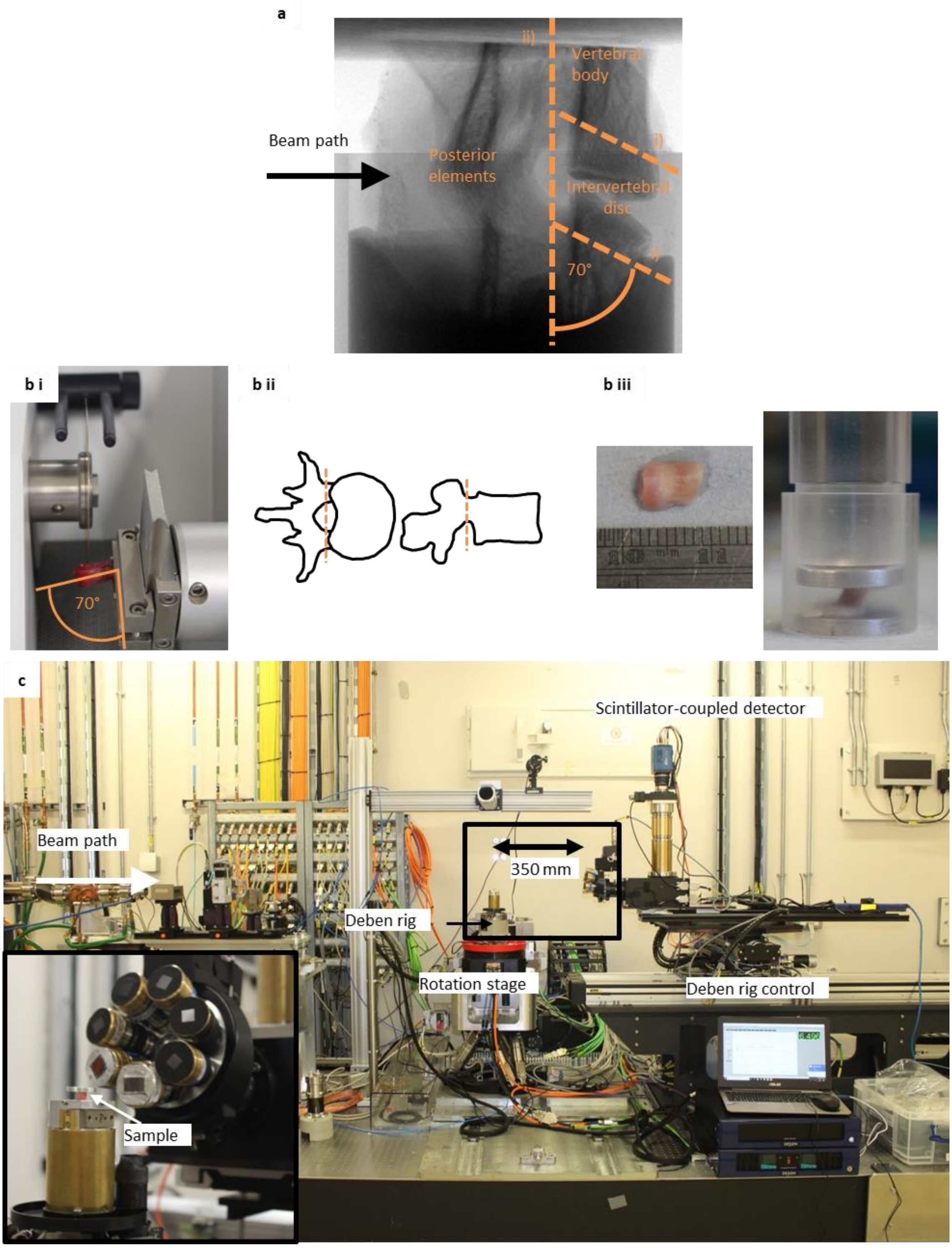

Suppl. Fig. S1: Sample preparation and beamline for sCT. a, *Radiograph of a spine segment. Orange lines show position of cuts made during sample preparation.* ***b,*** *Sample preparation step. Angled cuts were made (**i**), posterior elements were removed (**ii**) to limit calcified tissue in the beam*

*path and the segment set in the holder using epoxy resin (**iii**). **c,** Beamline set-up with Deben rig (open) and sample in place. A propagation (sample-scintillator) distance of 350 mm was used to provide decent in-line phase contrast.*

It is difficult to image at the soft-calcified tissue interface since the calcified endplate causes decreased signal: noise mainly caused by streaking artefacts (which can be severe with propagation distances suitable for soft tissues) which obscure features in the soft tissue[26]. In Suppl. Fig. S1a an x-ray projection image is shown through a spine segment where the dark areas indicate high x-ray absorption by calcified material such as the posterior elements and vertebral bodies. Removing the posterior elements and positioning the sample so that endplates were parallel with the beam (Suppl. Fig. S1) increased the number of photons reaching the disc structures in the field of view and decreased the variation in absorption in the beam path. This allowed us to resolve all lamellae and individual fibre bundles throughout the IVD (Figure 1, Suppl. Fig. 2). The signal- and contrast-to-noise ratios remained stable for all scans during the compression sequence (Suppl. Table 1). It was therefore possible to select different regions of interest for comparative analysis.

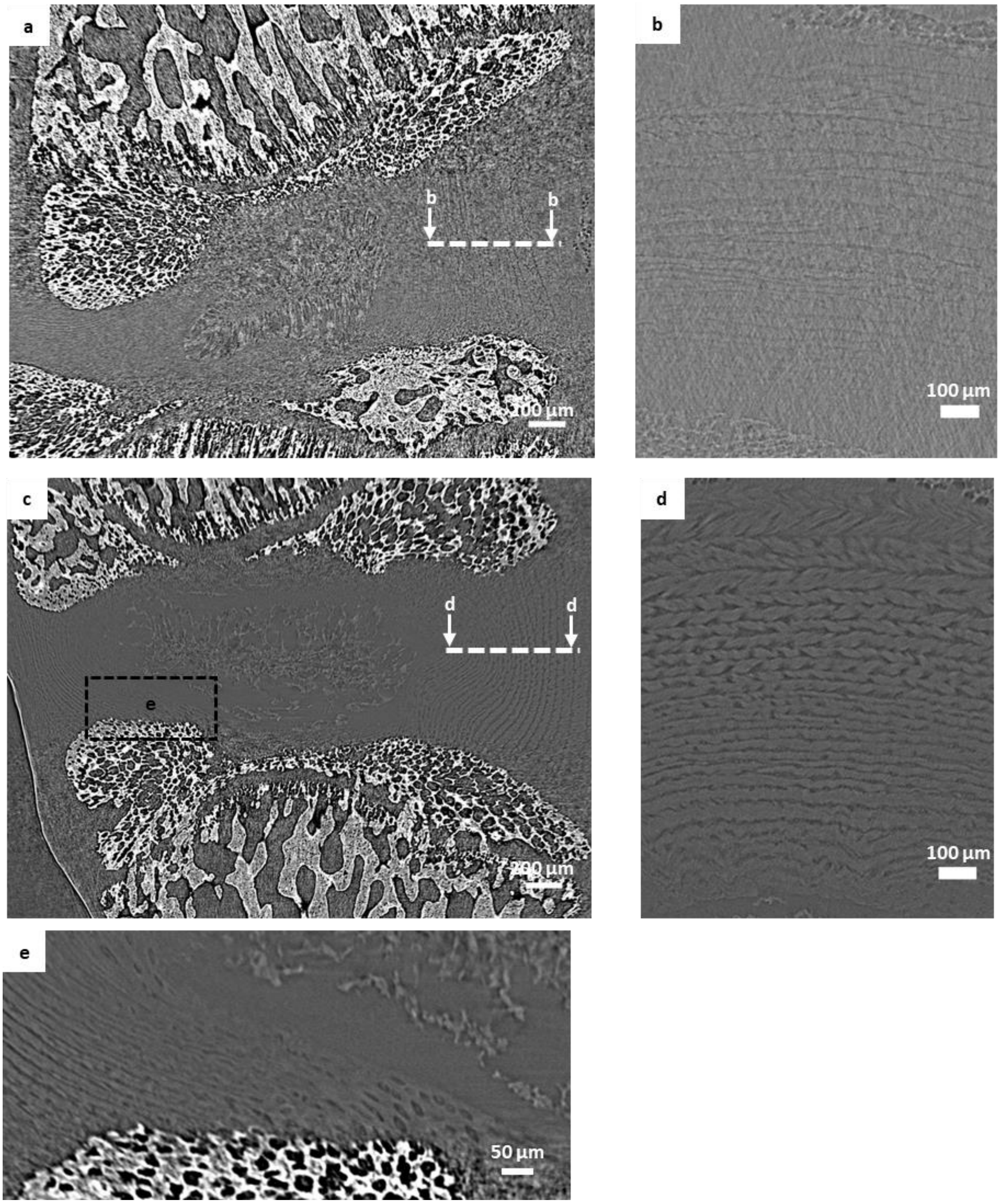

Suppl. Fig. S2: Example showing improvement in imaging. a, *previous attempt (a & b) at imaging intact intervertebral disc. There was low contrast to noise (0.32) and streaking due to highly absorbing calcified tissue in the beam path.* ***b,*** *anterior region. Image quality was improved for the study (c, d & e) with higher contrast to noise (2.27)* ***c,*** *Mid sagittal slice showed improved image quality where* ***d,*** *all lamellae were resolved.* ***e****, It was also possible to visualise the previously unresolved AF-endplate junction where cells can be seen in the cartilaginous endplate (Suppl. Video).*

Suppl. Table 1: SNR and CNR. *To quantify image quality signal-to-noise and contrast-to-noise ratios were calculated for the AF region in the images. Where signal-to-noise is calculated as mean grey value in the AF region divided by standard deviation of image noise (background; outside the sample) :* S_{af}/σ_0. *Contrast-to-noise ratio is given by the difference in mean AF image signal and noise signal, divided by standard deviation of image noise :* $(S_{af}-S_0)/\sigma_0$. *Calculations used 200 voxel isometric volumes.*

	Anterior		Posterior	
	SNR	CNR	SNR	CNR
First scan	2.27	1.76	2.00	1.49
Last scan	2.41	1.75	2.10	1.43

The increase in signal-to-noise from this imaging setup allowed shorter scan times by reducing the number of projections required. Previously 8001 projections were collected compared to 5001 in this study, 4021 (2π / sCMOS width) were required to avoid under-sampling; the excess helped increase signal:noise. This means that the samples were exposed to less radiation and scans are less likely to include movement artefacts from slow stress relaxation meaning that SNR was increased and remained stable between the first and last scans.

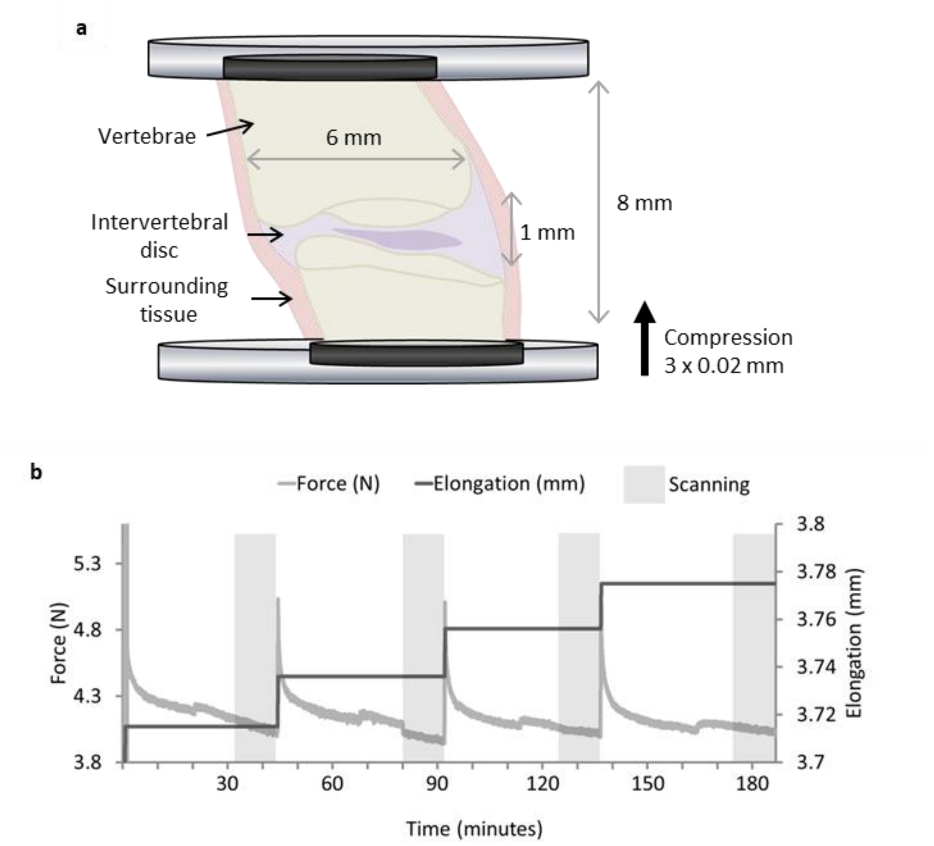

Suppl. Fig. S3: In situ imaging. a, *Rat spine segments were cemented into sample holders. Compression was from the bottom plate.* ***b,*** *Compression regime.1 N preload was applied and held, followed by a relaxation period and then the first scan. This was followed by 0.02 mm (~2% strain) compression, relaxation period and associated scan; repeated three times to obtain a full loading sequence.*

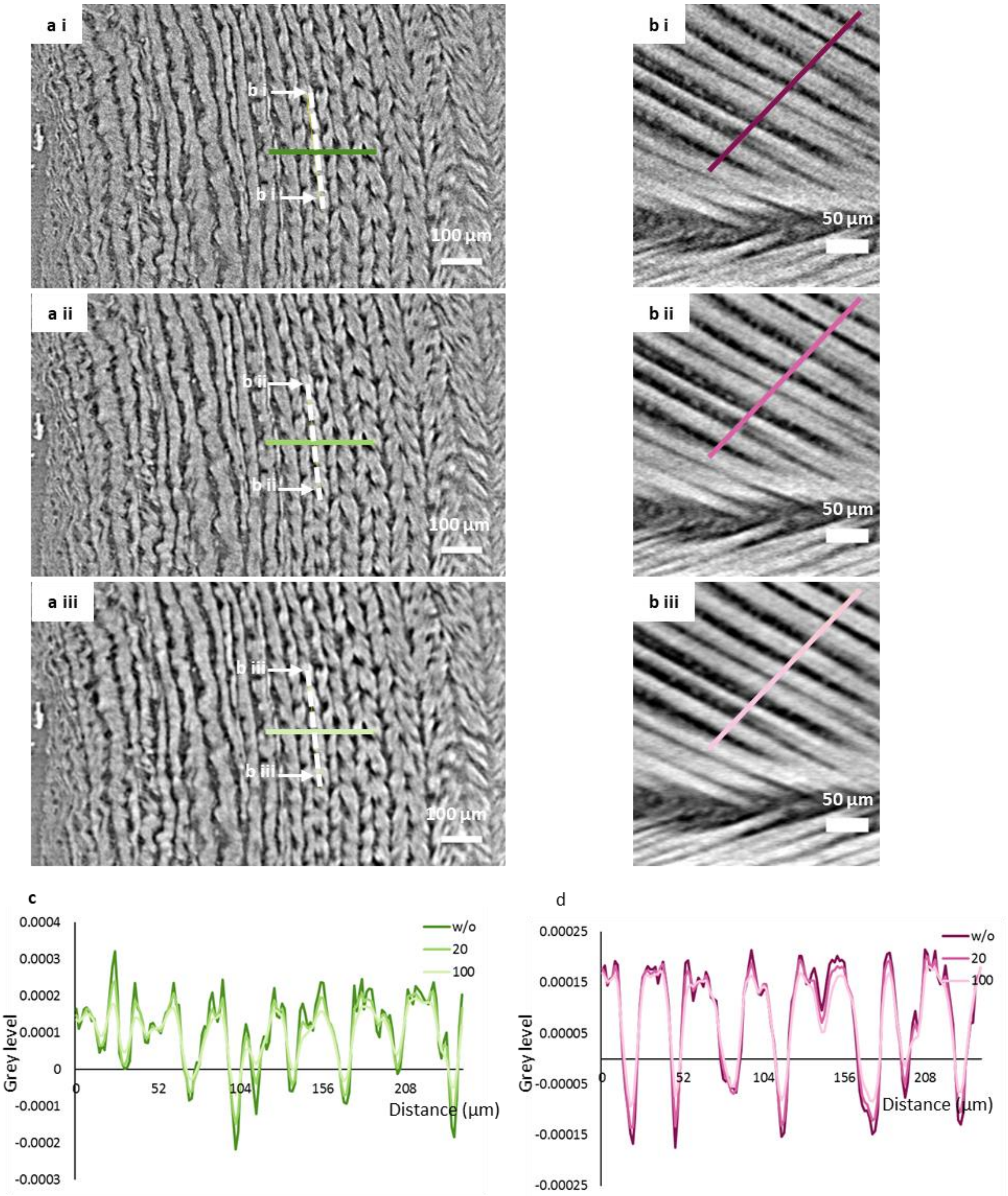

Suppl. Fig. S4: Reconstruction tailored for different analyses. a, *Transverse slice showing all anterior lamellae for reconstruction* ***ai,*** *without, and* ***aii,*** *with δ/β=20 and* ***aiii,*** *δ/β=100 phase retrieval.* ***b,*** *Radial slice showing fibre bundles for reconstruction* ***bi,*** *without, and* ***bii,*** *with δ/β=20 and* ***biii,*** *δ/β=100 phase retrieval.* ***c & d,*** *respective line profiles. Reconstruction with high phase retrieval (δ/β=100) is best used for visualisation purposes, low phase retrieval (20 δ/β) was used for fibre tracing, and reconstruction without phase retrieval was used to retain fibre image texture for digital volume correlation.*

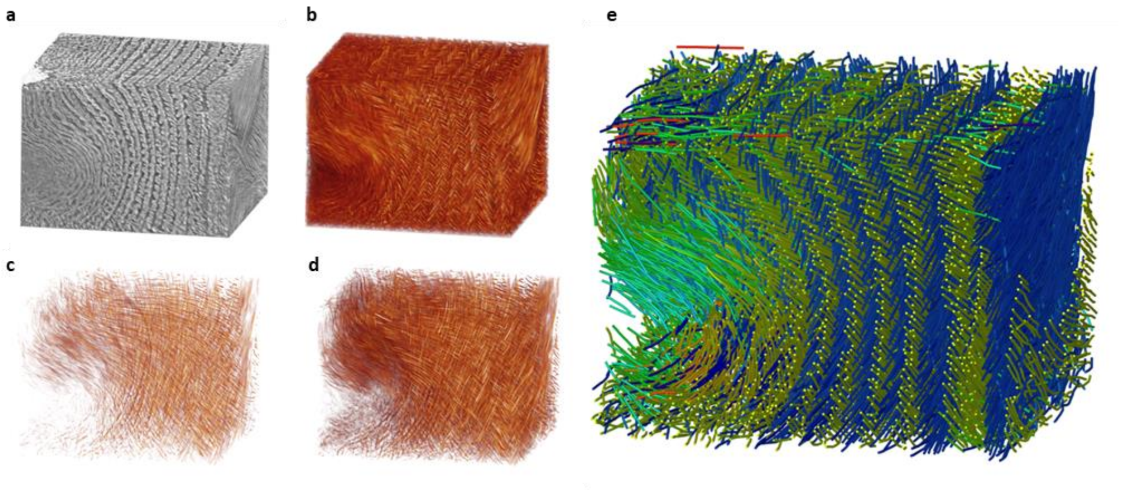

Suppl. Fig. S5: Fibre tracing in Avizo. a, *Render of anterior region of interest.* ***b,*** *Region of interest after cylinder correlation to enhance fibre-like structures results.* ***c,*** *High starting threshold for fibre tracing (100).* ***d,*** *Minimum threshold for fibre tracing continuation values (80).* ***e,*** *Traced fibres (Suppl. Video 1)*

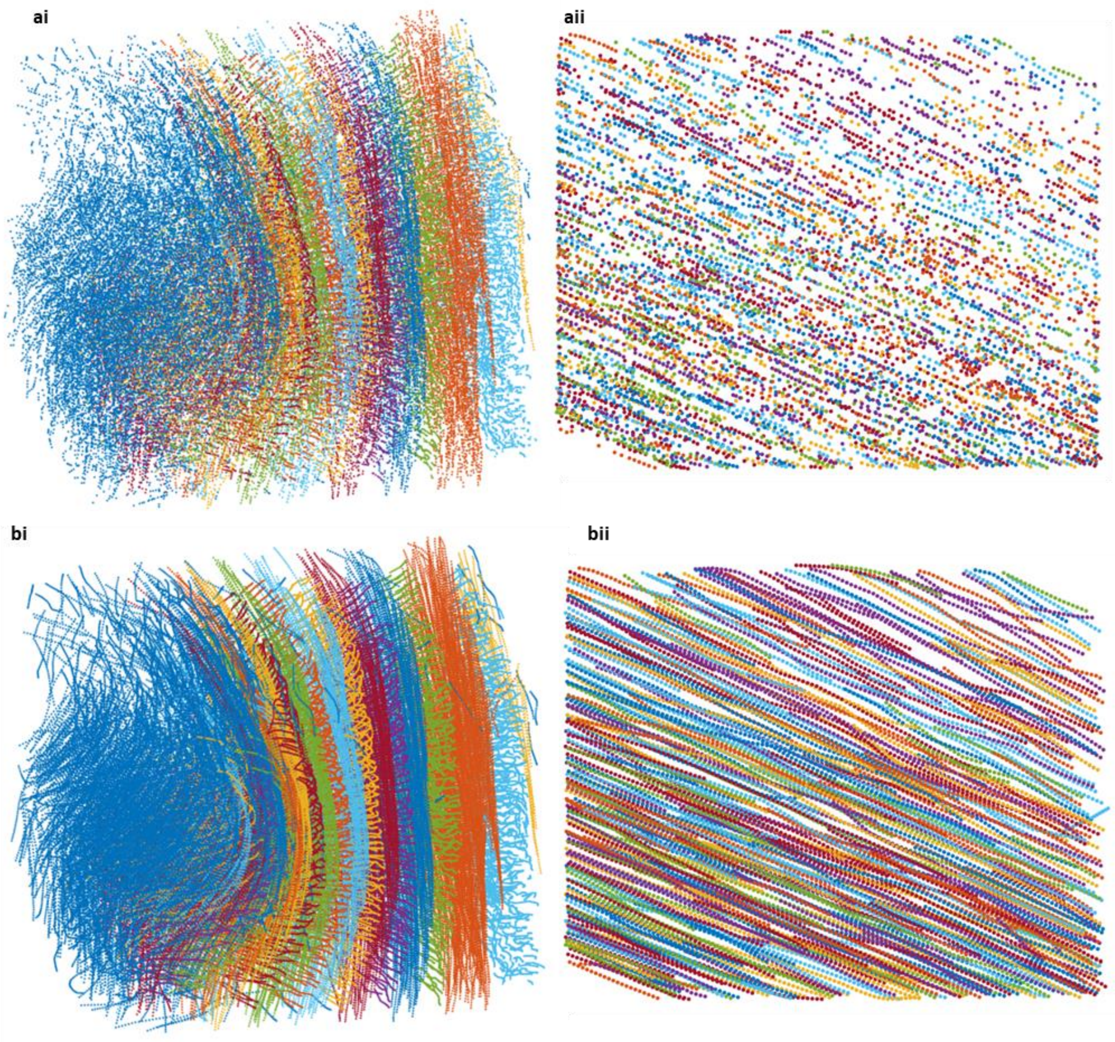

Suppl. Fig. S6: Example of anterior region point cloud seeding. a, *Points are not uniformly spaced along fibres when exported from Avizo fibre tracing (Suppl. Video 2) shown for ai, the whole region of interest labelled by lamellae and aii, in an example single lamella labelled by fibre.* ***b,*** *Points are seeded along each fibre using Matlab FibreTracing2PC.m function. This study used fibre point seeding at 5 pixel (8 µm) spacing (Suppl. Video 3) shown for bi, the whole region of interest labelled by lamellae and bii, in an example single lamella labelled by fibre.*

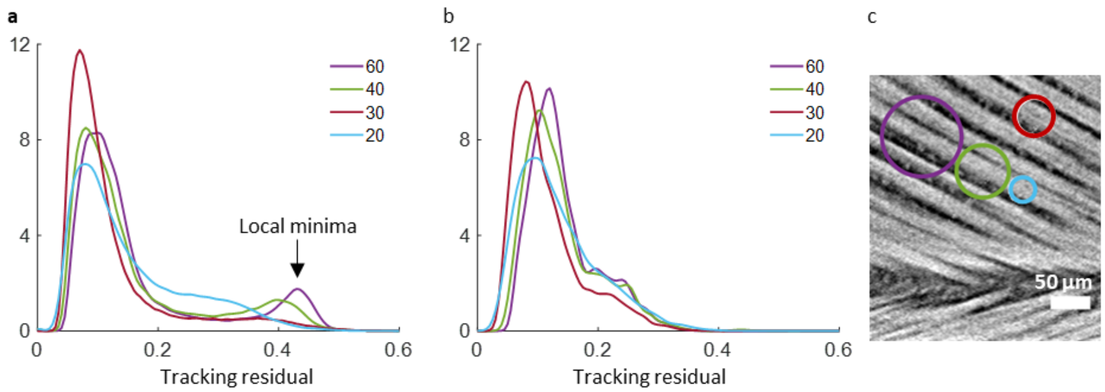

Suppl. Fig. S7: Tracking residual for sub volume size. a, *Anterior probability distribution of fibre DVC residual of sub volumes ranging from 60 voxels to 20 voxels. Larger sub volumes usually have lower tracking uncertainty however if the sample has repeating features such as fibres then local minima may be found. This is evident for larger sub volumes (60 & 40 voxels) where there is a secondary peak at higher tracking residual. Sub volume size of 20 voxels shows an increase in tracking uncertainty which may be due to insufficient image texture at this scale (see* ***c****, blue circle)* ***b,*** *Posterior-lateral probability distribution of fibre DVC residual for a range of sub volume sizes* ***c,*** *Example sub volume sizes. Red circle shows the chosen sub volume size (30). For the best results, the sub volume included the edges of the fibre but did not overlap into the neighbouring fibre.*

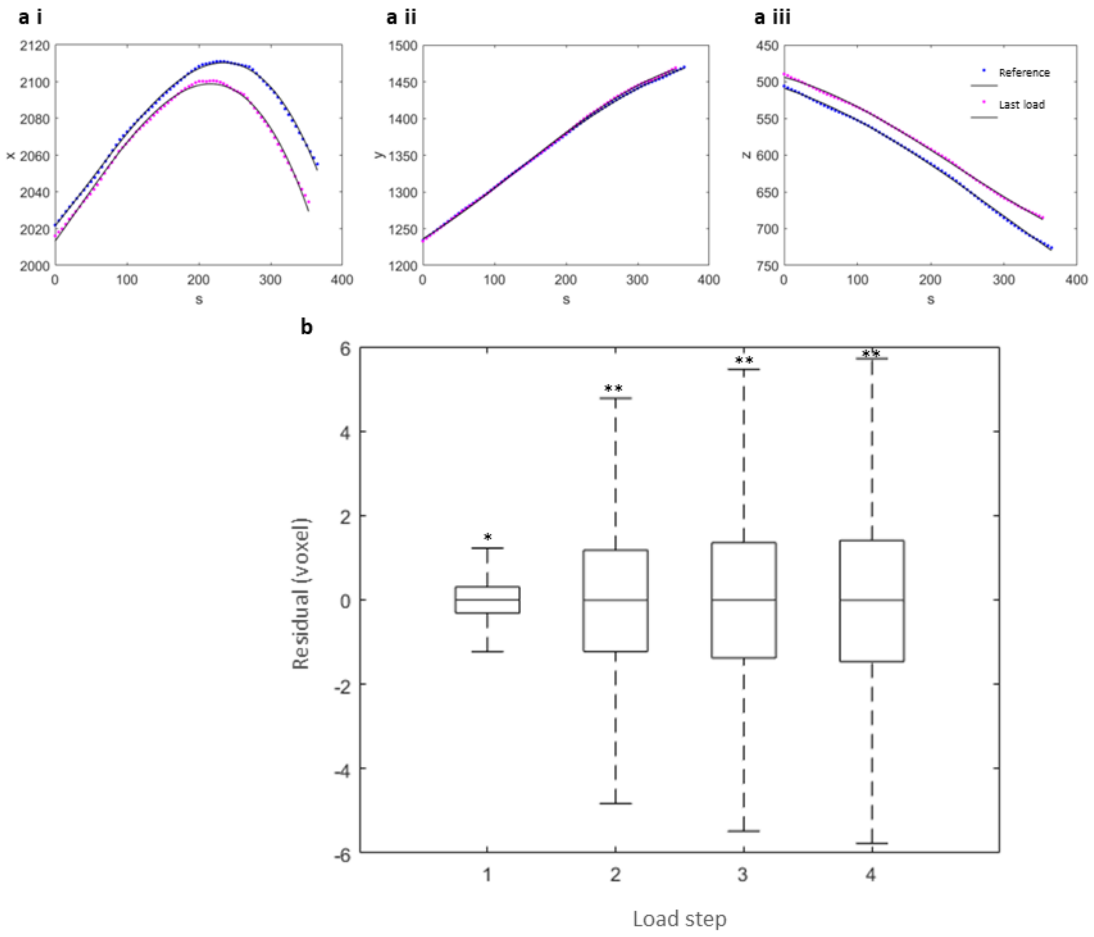

Suppl. Fig. S8: Space curve fitting of fibres. a, *Example of the fibre fitting process. Parametric set of third order polynomial, weighted by DVC residual, fitting was used. A single fibre from the reference (blue) and last load (magenta) step is shown as an example for* ***ai,*** *x* ***aii,*** *y* ***aiii,*** *z dimensions where s is distance along the fibre.* ***b,*** *Space curve fitting residual across load steps. * 99% of fibres had $R^2 > 0.9$, ** 97% of fibres had $R^2 > 0.9$.*

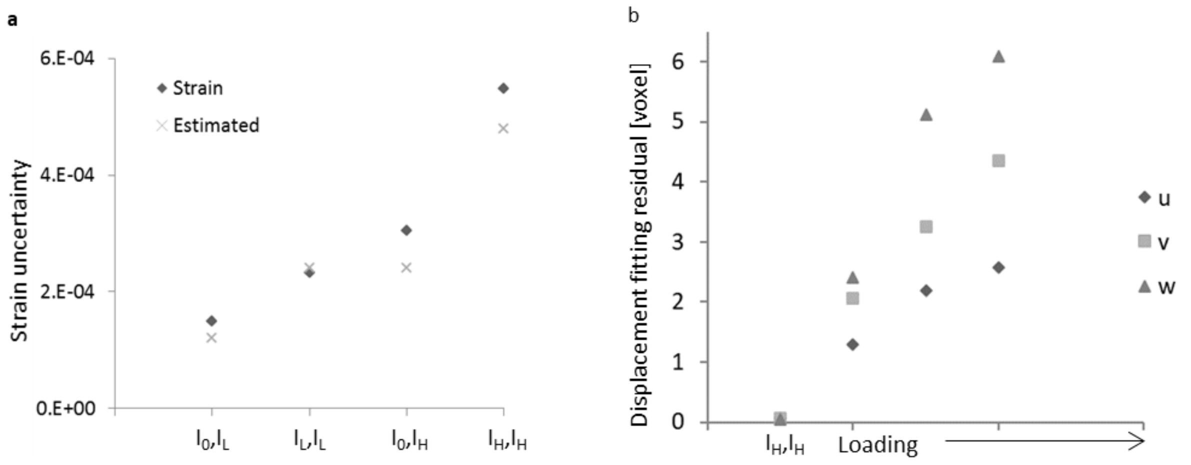

Suppl. Fig. S9 : Fibre DVC sensitivity to noise. *The same image volume with different levels of added noise were correlated to measure strain uncertainty and DVC robustness to image noise.* ***a,*** *Strain uncertainty (standard deviation) for increasing level of image noise. Four different cases were correlated. Where I_0 original image volume, I_L low level of noise, I_H high level of noise.* ***b,*** *Displacement fitting residual standard deviation. It is expected that there is an increase in displacement fitting residual as displacement is cumulative with each load step.*

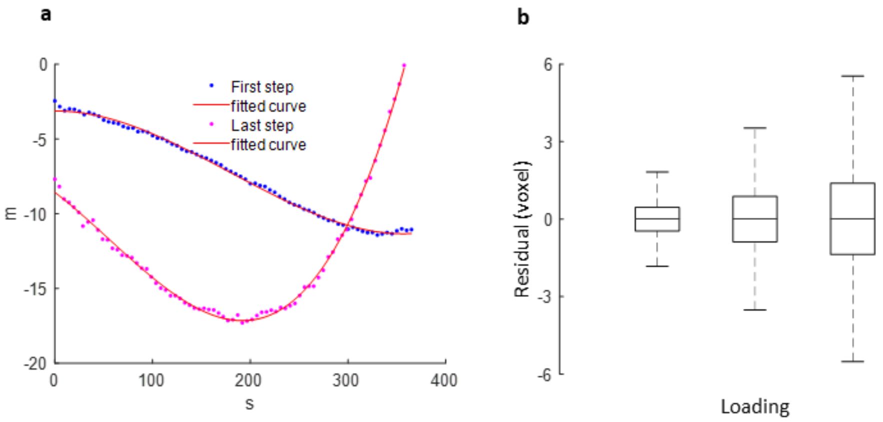

Suppl. Fig. S10: Goodness of fit for displacement values. a, *example of displacement fitting for a single fibre.* ***b,*** *fitting residual for anterior region for all load steps. Over 77% of the fibres had $R^2 >$ 0.8. N.B. fitting process is weighted by DVC tracking accuracy and so it is expected to have increasing residual with loading as tracking noise is added with each step. Inspecting fibres such as above shows the fitting process has a smoothing effect.*

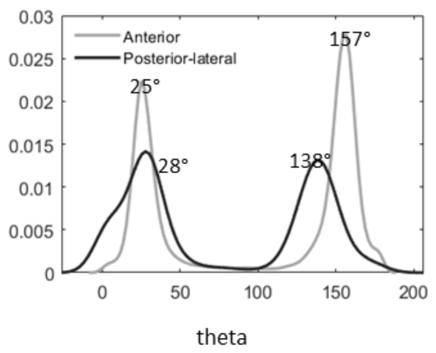

Suppl. Fig. S11: Regional difference in fibre orientation. *Bimodal probability distribution peaks are closer for posterior-lateral fibres indicating a steeper incline. Pitch difference of 11° ((157-25)-(138-28))/2*

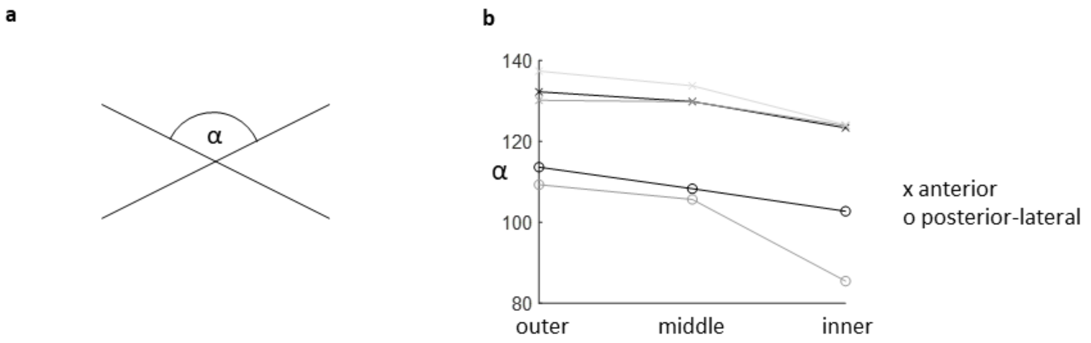

Suppl. Fig. S12: Orientation repeats. a, *angle α measured for comparison between samples.* ***b,*** *Difference of bimodal peaks in each region plotted for repeat samples. α decreases with depth; steeper fibres in inner lamellae.*

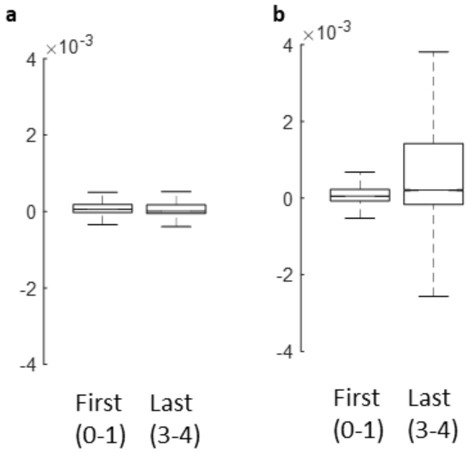

Suppl. Fig. S13: Change in curvature for first and last load steps. a, *Anterior.* ***b,*** *Posterior-lateral*

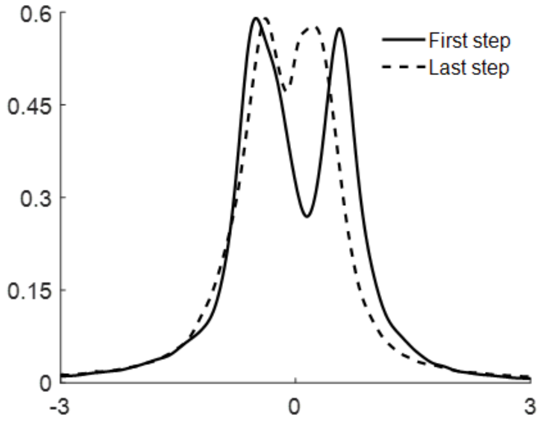

Suppl. Fig. S14: Re-orientation repeat. *Peaks are closer together with increasing load meaning that re-orientation of the fibres decreased with load.*

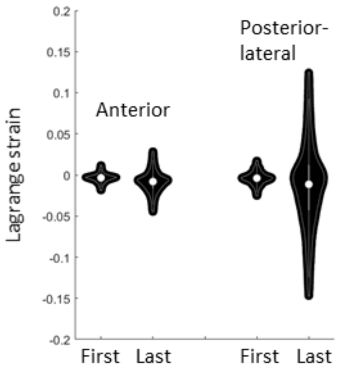

Suppl. Fig. S15: Fibre strain for first and last load steps.
